# Prosaposin orchestrates a TGFβ1-driven paracrine loop between Schwann cells and gastric cancer to accelerate perineural invasion

**DOI:** 10.1186/s13046-026-03652-3

**Published:** 2026-01-24

**Authors:** Shijie Yang, Huan Xi, Miao Yu, LinFan Qi, Lin Ma, ZiJian Wu, Guangming Zhang, Shixun Ma, Hui Cai

**Affiliations:** 1 The First Clinical Medical College of Lanzhou University, Lanzhou, Gansu 730000 China; 2 Gansu Key Laboratory of Molecular Diagnostics and Precision Medicine for Surgical Oncology, Gansu Provincial Hospital, Lanzhou, Gansu 730000 China; 3 The Second Hospital & Clinical Medical School, Lanzhou University, Lanzhou, Gansu 730000 China; 4 Third Clinical Medical College of Lanzhou University, Lanzhou University, Lanzhou, Gansu 730000 China; 5 Department of Phase Ⅰ Clinical & Research Ward, Gansu Provincial Hospital, Lanzhou, Gansu 730000 China; 6 NHC Key Laboratory of Diagnosis and Therapy of Gastrointestinal Tumor, Gansu Provincial Hospital, Lanzhou, Gansu 730000 China; 7 Gansu University of Traditional Chinese Medicine, Lanzhou, Gansu 730000 China; 8 Department of General Surgery Clinical Center, Gansu Provincial Hospital, Lanzhou, 730000 China

**Keywords:** Gastric cancer, Schwann cells, Perineural invasion (PNI), PSAP

## Abstract

Perineural invasion (PNI) is an established adverse prognostic factor in gastric cancer (GC), yet the molecular events initiating this process remain poorly defined. In this study, we identify Schwann cells (SCs) as active facilitators of PNI and elucidate a reciprocal signaling axis between GC cells and SCs that promotes PNI. Using a GC–SCs co-culture model, we show that SCs enhance PNI potential in GC cells, accompanied by increased expression of prosaposin (PSAP), a lysosomal secretory protein, in both co-cultured cells and PNI-positive tumor specimens. Mechanistic studies reveal that PSAP forms a complex with cathepsin D (CTSD) and galactocerebrosidase (GALC) to inhibit autophagy and to promote PNI progression. GC-derived PSAP activates the G protein–coupled receptor 37(GPR37) on SCs, initiating RAC1-dependent cytoskeletal remodeling. This activation also induces secretion of transforming growth factor-β-1(TGFβ1) by SCs, which, in turn, binds transforming growth factor-β receptor-II (TGFβRII) on GC cells and activates a TGFβ1/Smad4/Sortilin signaling cascade. This pathway amplifies PSAP production and reinforces tumor–nerve interactions, establishing a feedforward paracrine loop that drives PNI. Functionally, enforced PSAP expression in GC cells significantly enhances PNI both in vitro and in vivo. Clinically, co-expression of PSAP, TGFβ1, and SCs marker S100β correlates with PNI incidence and improve PNI discrimination. Collectively, these findings define a novel PSAP–TGFβ1–Sortilin axis that mediates SCs–tumor crosstalk and sustains PNI in GC. Disrupting this paracrine loop may provide a therapeutic avenue to limit PNI and improve outcomes.

**Graphical Abstract:**

This schematic model illustrates a self-amplifying tumor–nerve signaling circuit between GC cells and SCs that promotes PNI by coupling intracellular PSAP sorting to paracrine communication. PSAP is overexpressed in GC cells. The 65 kDa PSAP synthesized in the endoplasmic reticulum (ER) is transported to the Golgi and sorted into two trafficking routes. A fraction binds Sortilin in the trans-Golgi network (TGN) and is delivered to lysosomes, during which PSAP forms a complex with CTSD/GALC to inhibit autophagy and potentiate malignant traits. In parallel, another fraction is processed and secreted as a 75 kDa form, which engages GPR37 on SCs, activates RAC1-dependent cytoskeletal remodeling, and induces TGF-β1 secretion. SC-derived TGF-β1 signals back to GC cells via the TGF-β1/Smad4 pathway to repress Sortilin expression, reducing PSAP–Sortilin interaction and limiting lysosomal targeting, which in turn increases PSAP abundance and secretion. Collectively, these events establish a feed-forward paracrine amplification loop that sustains tumor–nerve crosstalk and accelerates PNI.

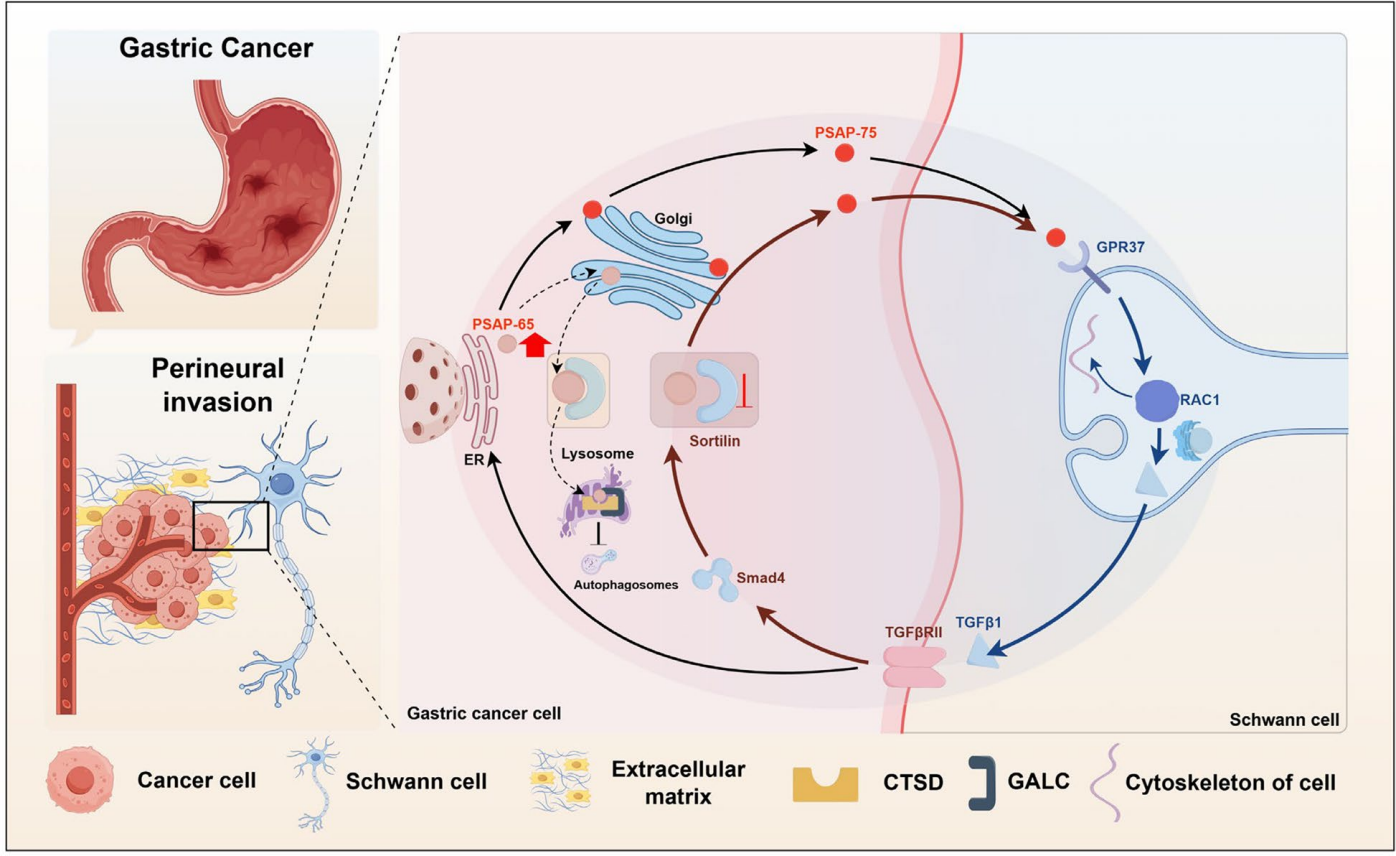

**Supplementary Information:**

The online version contains supplementary material available at 10.1186/s13046-026-03652-3.

## Introduction

GC ranks as the fifth most prevalent malignancy and the fourth leading cause of cancer-related mortality globally [1]. China bears a disproportionately high burden of GC, with an annual incidence of 357,000 new cases and 260,400 deaths, exhibiting incidence and mortality rates exceeding twice the global average [2]. Despite significant advances in multimodal therapies—including surgical resection, chemotherapy, and immunotherapy—the inherent invasive and metastatic properties of tumor cells remain critical barriers to therapeutic efficacy. The 5-year overall survival rate for GC patients worldwide remains approximately 20%, with metastatic cases demonstrating even more dismal prognoses [3], underscoring the urgent need to elucidate the pathological mechanisms driving GC progression.

PNI, a histopathological hallmark frequently observed in clinical GC specimens [4], has emerged as an independent prognostic indicator in multiple oncology guidelines, often guiding adjuvant chemotherapy decisions post-resection [5, 6]. Clinical cohort studies consistently associate PNI with aggressive clinicopathological features, including undifferentiated/diffuse histology, deeper tumor invasion, lymph node metastasis, advanced staging, and reduced survival [7, 8]. Notably, tumors exhibiting enhanced neural innervation demonstrate heightened invasiveness and metastatic potential [9, 10]. Intriguingly, partial vagal denervation has been shown to reduce gastric tumorigenesis in preclinical models [11–13], while PNI itself may serve as a distinct metastatic pathway beyond conventional dissemination routes [14].

Pathologically, PNI manifests as tumor cell infiltration along nerve trunks [15], reflecting dynamic bidirectional interactions between malignant cells and neural components [16]. While SCs are recognized as pivotal regulators of the perineural microenvironment—through secretion of cytokines, growth factors, and neurotrophic factors, as well as direct tumor cell contact [17–20]—the precise initiator signals and cellular crosstalk mechanisms underlying GC-associated PNI remain poorly defined. To address this knowledge gap, we established a GC cells-SCs coculture system and performed proteomic profiling, identifying prosaposin (PSAP), a lysosomal protease secreted by GC cells, as a critical mediator of SCs activation, axonal regeneration, and PNI initiation.

Current research on GC-PNI molecular regulation remains fragmented, particularly regarding SCs-specific signaling networks. We revealed that GC-derived PSAP operates via paracrine signaling to modulate SCs functionality, subsequently triggering TGFβ1 secretion and PNI progression. Through comprehensive in *vitro* and in *vivo* experimentation combined with clinical validation using GC tissue specimens, we delineated a PSAP-driven crosstalk axis between SCs and GC cells. In our cohort, higher PSAP expression in GC tissues was positively associated with the severity of PNI (*p* < 0.001), indicating that PSAP may serve as a candidate predictive biomarker and a plausible therapeutic target. Collectively, these data advance our understanding of the molecular underpinnings of GC-associated PNI and may inform the development of PSAP-oriented strategies for prognostication and therapeutic intervention.

## Materials and methods

### Patients and clinical samples

This study included two independent cohorts. The total of 104 patients with gastric adenocarcinoma who underwent radical gastrectomy and were confirmed by pathology in the main campus of Gansu Provincial People's Hospital from January 2018 to January 2019 were collected. Due to incomplete medical records and loss of follow-up, 75 patients were finally included in the training cohort. The validation cohort consisted of 93 patients with gastric adenocarcinoma who underwent radical gastrectomy at the Lanzhou New District branch of Gansu Provincial’s Hospital (Lanzhou New District People’s Hospital) and the West District of Gansu Provincial’s Hospital between October 2017 and October 2025. None of the patients received neoadjuvant therapy (chemotherapy, radiotherapy or immunotherapy) prior to surgery. Archived pathological specimens and clinicopathological data (including age, gender, TNM staging, and PNI status) were collected. The study protocol was approved by the Ethics Committee of Gansu Provincial People’s Hospital (approval No. 2024–630), and the study was conducted in accordance with the Declaration of Helsinki. Follow-up was performed by telephone from 30 October 2024 to 30 November 2025 to ascertain overall survival, recurrence status and disease-free survival; patients were censored at the date of last successful contact.

### Cell lines and culture conditions

Human GC cell lines: AGS (CL-0022), MKN-45 (CL-0292), and HGC-27 (CL-0107), along with rat Schwann cells RSC96 (CL-0199), were obtained from Procell Life Science & Technology Co., Ltd (Wuhan, China). Cells were maintained as follows:1. AGS: Ham's F-12 + 10% Fetal bovine serum (FBS) + 1% penicillin/streptomycin (P/S);2. MKN45/HGC27: RPMI-1640 + 10% FBS + 1% P/S;3. RSC96: DMEM + 10% FBS + 1% P/S. All cultures were incubated at 37 °C in a humidified 5% CO₂ atmosphere. Cryopreservation utilized 55% basal medium + 40% FBS + 5% Dimethyl sulfoxide (DMSO).

### Co-culture system and conditioned media

A 0.4-μm pore Transwell system (Corning, USA, #3450) was employed for co-culture experiments. GC cells (4 × 10^5^) were seeded in 6-well plates, with RSC96 cells (4 × 10^5^) in upper chambers. After 48 h co-culture, conditioned media (CM) were collected by centrifugation (2,000 × g, 10 min) and stored at −80 °C.

### Proteomic profiling

#### Sample preparation

Cell lysates were homogenized in SDS buffer (4% SDS, 100 mM Tris–HCl, pH 7.6) containing 1% DTT, followed by 5-min ice-bath sonication (Xinzhi, China, JY92-IIN). Proteins were alkylated with 55 mM iodoacetamide, precipitated with acetone, and resuspended in 8 M urea/100 mM TEAB (pH 8.5). High-abundance serum proteins were depleted using ProteoMiner® beads (Bio-Rad, USA, # 1633006).

#### LC–MS/MS analysis

Trypsin-digested peptides (20 μg) were separated on a PepMap™ Neo C18 column (150 μm × 15 cm, 2 μm; Thermo Fisher, USA, # DNV75150PN) using a 22.6-min acetonitrile gradient (4–55%, 0.1% formic acid) via Vanquish Neo UHPLC. Data-independent acquisition (DIA) was performed on an Orbitrap Astral mass spectrometer (Thermo Fisher, USA) with 300 variable windows (m/z 380–980, HCD at 25% NCE).

#### Bioinformatics

Raw data were processed using DIA-NN v1.8 against UniProt Human database (2023_01 release). Differential expression was defined as |log₂ (fold change)|> 1 with *p* < 0.05 (two-tailed t-test). Functional annotation used InterProScan v5.52; protein networks were built with STRING DB v11.5.

### Co-Immunoprecipitation (Co-IP) and mass spectrometry

#### Co-IP protocol

Cells were lysed in ice-cold Co-IP buffer (20 mM Tris–HCl pH 7.4, 150 mM NaCl, 1% NP-40) supplemented with 1 × protease inhibitor cocktail (Roche, Switzerland, #4,693,159,001) using 30-min orbital shaking at 4 °C. Lysates were clarified by centrifugation (12,000 × g, 10 min, 4 °C). Protein G agarose beads (50 μL/sample; Merck,Gemany,#GE17-0618–01) were pre-washed with Co-IP buffer and divided into three groups: 1. Pre-cleared group: Incubated with lysates for 4 h at 4 °C to remove nonspecific binding;2. IgG control: Coated with 10 μg rabbit IgG (Cell Signaling Technology, USA, #2729);3. Experimental group: Coated with 10 μg anti-PSAP antibody (Abcam, England, #ab308122).

Pre-cleared lysates were incubated with antibody-conjugated beads for 4 h at 4 °C. After four washes with Co-IP buffer, bound complexes were eluted using 2 × Laemmli buffer (Bio-Rad, USA, #1,610,737) with 5-min boiling at 95 °C.

#### Mass spectrometry sample preparation

Co-IP eluates were separated on 10% SDS-PAGE gels (80 V for 20 min, then 120 V for 90 min). Protein bands were visualized by Coomassie Brilliant Blue R-250 staining (Bio-Rad, USA, #1,610,436). Target bands were excised, destained, and subjected to 1. Reduction: 10 mM DTT (56 °C, 30 min); 2. Alkylation: 55 mM iodoacetamide (RT, 20 min in darkness);3. Trypsin digestion: Sequencing-grade trypsin (Promega, USA, #V5111; 37 °C, 16 h);4. Peptides were desalted using C18 StageTips (Thermo Fisher, USA, #SP301) and lyophilized.

#### LC–MS/MS analysis

Peptides were separated on a nanoElute UHPLC system (Bruker) equipped with australis™ C18 column (75 μm × 25 cm, 1.6 μm; IonOpticks, Australian, #AUR2-25075C18A). Gradient conditions: 5–35% acetonitrile/0.1% formic acid over 90 min. Data acquisition used DDA data-dependent acquisition on timsTOF Pro 2 (Bruker) with following parameters:1. MS1: m/z 100–1700, 1/k0 0.6–1.6 Vs/cm^2^;2. MS2: Top*N =* 10, dynamic exclusio*n =* 30 s.

#### Bioinformatics analysis

Raw files were processed with MaxQuant (v2.1.3) against UniProt Human proteome database (2023_01 release). Search parameters included:1. Fixed modification: Carbamidomethylation (C);2. Variable modifications: Methionine oxidation, N-terminal acetylation;3. FDR threshold: 1% at PSM/protein levels. Interaction Screening Criteria: Proteins showing > twofold enrichment vs IgG control (Fisher's exact test, *p* < 0.05) were considered specific interactors.

### Surface Plasmon Resonance (SPR) assay

Surface plasmon resonance analyses were conducted on a Biacore S200 system (GE Healthcare) with a CM5 sensor chip to characterize the interaction between PSAP and GPR37. Prior to ligand coupling, the chip surface was activated by sequential injection of 0.4 M EDC and 0.1 M NHS, generating reactive ester groups for subsequent covalent immobilization. PSAP was diluted in 10 mM sodium acetate buffer (pH 4.0) and introduced onto the activated surface to achieve stable attachment, after which residual esters were quenched with 1 M ethanolamine (pH 8.5). All binding measurements were performed using PBS supplemented with Tween-20 and DMSO as the running buffer to ensure signal stability. Increasing concentrations of GPR37 were then injected to monitor association with immobilized PSAP, followed by dissociation under continuous buffer flow; each cycle consisted of approximately 60 s of association and 90 s of dissociation. Depending on the tolerance of the immobilized PSAP, the sensor surface was regenerated using either 10 mM glycine–HCl (pH 3.0) or 50 mM NaOH to restore baseline conditions. Reference-subtracted sensorgrams were subsequently analyzed using the Biacore evaluation software, and kinetic parameters—including the equilibrium dissociation constant (Kd)—were obtained by fitting the binding curves to a 1:1 Langmuir interaction model.

### Chromatin Immunoprecipitation (ChIP)–qPCR

ChIP experiments were undertaken using the CST ChIP kit (CST, USA, # 9005), with all procedures carried out under conditions recommended by the supplier. Cells were crosslinked to preserve protein–DNA interactions, followed by cell disruption and controlled chromatin shearing. The fragmented chromatin was subsequently incubated with the designated antibodies to capture target-associated DNA regions. After immunocomplex isolation and reversal of crosslinks, purified DNA was subjected to quantitative PCR analysis. Relative enrichment levels were determined using the %Input approach, and the oligonucleotides employed in these assays are listed in Supplementary Table 1–2.

### Dual-luciferase reporter assay

For luciferase reporter measurements, cells were transfected after attachment with the Sortilin promoter–luciferase plasmid together with the Smad4 overexpression (OE) vector using Lipofectamine 3000. Control conditions consisted of a non-promoter construct (NC) and an empty backbone vector. At 48 h post-transfection, luciferase activity was quantified using a dual-luciferase detection system (Thermo Fisher Scientific, USA, # 16,185) and read on a BioTek microplate detector. Data were processed by normalizing firefly luminescence against Renilla signals from the same wells.

### mRFP-GFP-LC3B adenoviral assay

Cells were infected with the mCherry-GFP-LC3B adenovirus (Beyotime, Shanghai, China; #C3011) and cultured for 48 h to allow expression of the reporter. After washing with PBS, cells received the indicated treatments and were examined by confocal microscopy. In this dual-fluorescent LC3 system, GFP fluorescence is quenched in acidic autolysosomes whereas mRFP remains detectable; thus, yellow puncta mark autophagosomes and red puncta indicate autolysosomes. Autophagic flux was evaluated by counting the respective puncta.

### Functional assays

#### RNA interference and plasmid transfection

Lentiviral particles expressing human, Small interfering(si) RNA, short hairpin (sh) RNA and Plasmid were purchased from GenePharma (Shanghai, China) and Tsingke (Beijing, China) (Supplementary Table 1). Transient transfection was performed using Lipofectamine™3000 (Invitrogen, USA, #L3000015) according to the manufacturer's protocol. Knockdown (KD) was achieved by lentivirus-mediated shRNA transduction (cloned into the LV16 vector [U6 promoter/luciferase-puromycin]), whereas overexpression was achieved by LV5 vector (EF-1α promoter/GFP-puromycin) (both from GenePharma). The interference effects are shown in Supplementary Fig. 1A.

#### Cell proliferation analysis

Cells were seeded in 96-well plates at a density of 2,000 cells/well. After 24 h, Cell Counting Kit-8(CCK8) (APExBIO, USA, #K1018) was added, followed by incubation at 37 °C for 1 h. Absorbance was measured at 450 nm using a microplate reader.

#### Wound healing assay

Cells grown to 80–90% confluency in 6-well plates were scratched with a sterile pipette tip. Wound closure was monitored at 0, 24, and 48 h using an Olympus IX73 microscope (Japan). Migration rates were calculated as previously described [21]. Prior to scratch assays, culture conditions were optimized using high-adhesion 6-well plates (Corning, USA, #354,400), which significantly enhanced cell adhesion and promoted more stable and uniform monolayer formation.

#### Transwell invasion assay

Transwell chambers (8-μm pore size; Corning, USA, #3422) were pre-coated with 250 μg/mL Matrigel (BD Biosciences, USA, #356,234). Cells (1 × 10^4^) in serum-free medium were added to the upper chamber, while complete medium was placed in the lower chamber. After 48 h, non-invaded cells were removed, and invaded cells were fixed with methanol, stained with 0.1% crystal violet (Meryer, China, #C8470), and counted under a microscope.

#### Colony formation assay

Cells (1 × 10^3^/well) were seeded into 6-well plates and cultured for 14 days with medium replacement every 48 h. Colonies were fixed with 4% paraformaldehyde, stained with 0.5% crystal violet (Meryer, China, #C8470), and quantified.

### Animal models

All procedures were approved by Lanzhou University Animal Ethics Committee (approval No. MECI20240011) and complied with ARRIVE guidelines.

#### Dorsal Root Ganglion (DRG) isolation and co-culture

DRGs were isolated from 7-day-old male *BALB/c mice* under sterile conditions. Briefly, after excess carbon dioxide aspiration, dorsal root ganglia were surgically excised from vertebral columns using micro-dissection forceps and mechanically cleaned of surrounding nerve sheaths. Isolated DRGs were embedded in 20 μL growth factor-reduced Matrigel (Corning, USA, #356,231;8–12 mg/mL protein concentration) within 12-well plates (polymerized at 37 °C for 30 min). Cultures were maintained in neurobasal medium supplemented with 10% FBS, and 1% P/S at 37 °C/5% CO₂. Morphological changes were documented daily for 7 days using phase-contrast microscopy (Olympus IX83, Japan,10 × objective). Sciatic nerve function and the index of sciatic nerve function were measured weekly as described in the study published by Gil Z et al. [22].

#### DRG-PNI model

GC cells (5 × 10^4^) embedded in Matrigel were co-cultured with mouse DRGs. Nerve invasion index (α/γ ratio) was quantified microscopically [23, 24].

#### Sciatic nerve model

HGC27 variants (oePSAP/shPSAP) or cell mixtures were injected into sciatic nerves of BALB/c nude mice (*n =* 3/group). Neurological function was assessed weekly using sciatic functional index.

### Western Boltting (WB)

Total protein extraction was conducted using RIPA lysate (Boster Biological Technology, China, #AR0105). The procedure of WB was conducted in strict accordance with the established protocol of the manufacturer. GAPDH and Tublin were utilised as endogenous references. Primary and secondary antibodies are referred to Supplementary Table 2.

### ELISA

Secreted PSAP and TGFβ1 levels in cell supernatants were quantified using Human PSAP (CUSABIO, China, #CSB-E12837h) and Rat TGFβ1 (JIANGLAI BIO, China, # JL12342,) ELISA kits. Sample collection: Cells (AGS, MKN45, HGC27, RSC96) were cultured in Serum free medium for 24 h. Supernatants were centrifuged (2,000 × g, 10 min) to remove debris. Assay procedure:1. 100 μL standards/samples per well, incubated at 25 °C for 2.5 h; 2. Wash steps: 3 × with PBS + 0.05% Tween-20 (PBST); 3. 100 μL biotinylated detection antibody (1:100), 1 h at 25 °C; 4. 100 μL streptavidin-HRP (1:1,000), 45 min at 25 °C; 5. 100 μL TMB substrate, 30 min incubation in darkness; 6. Reaction stopped with 50 μL 2 N H₂SO₄. Absorbance at 450 nm was measured using a SpectraMax i3x microplate reader (Molecular Devices).

### Immunohistochemistry (IHC)

Formalin-fixed paraffin-embedded (FFPE) tissues were sectioned (4 μm), deparaffinized with xylene, and rehydrated through graded ethanol. Antigen retrieval was performed in citrate buffer (10 mM, pH 6.0) using microwave heating (20 min at 95 °C). Endogenous peroxidase activity was blocked with 3% H₂O₂ (Sigma, Gemmy, #H1009) for 10 min. Sections were incubated with primary antibodies (Supplementary Table 2) diluted in 3% BSA/PBS overnight at 4 °C, followed by HRP-conjugated secondary antibodies (1:500; Servicebio, China, #GB23303) for 1 h at 25 °C. Signals were developed with DAB substrate (Dako, Denmark,#K3468) and counterstained with hematoxylin. Images were acquired using an Olympus BX53 microscope (10 ×/40 × objectives).

### Multiplex Immunofluorescence Staining(mIHC)

#### TSA-based sequential staining

FFPE sections underwent five iterative staining cycles using tyramide signal amplification (TSA). Each cycle included:1. Primary antibody(Supplementary Table 2) incubation: Overnight at 4 °C; 2. Secondary antibody: HRP-conjugated (1:500, Servicebio,China,#GB23303); 3. Fluorescent tyramide: iF488/CY3/iF594/iF647-Tyramide (1:500; Servicebio,China, #G1231/G1223/G1242/G1232) incubated for 10 min in darkness; 4. Antibody stripping: Microwave treatment in citrate buffer (pH 6.0, 121 °C, 10 min).

#### Imaging analysis

Sections were counterstained with DAPI (Servicebio, China, #G1012) for 10 min and mounted with anti-fade medium (Servicebio, China, #G1401). Fluorescence images were captured using a Nikon Eclipse C1 microscope with following parameters: DAPI: Ex/Em 358/461 nm; FITC: Ex/Em 488/525 nm; CY3: Ex/Em 550/570 nm; iF594: Ex/Em 590/617 nm; CY5: Ex/Em 650/670 nm. Whole-slide scanning was performed on a 3DHISTECH Pannoramic MIDI (40 × resolution).

### Pattern drawing

The drawing of all pattern diagrams in this paper is completed through Figdraw (https://www.figdraw.com).

### Statistical analysis

All analyses were conducted following the *Guidelines for Reporting Statistical Analyses in Biomedical Research* (JAMA Network Open, 2022). Experimental data processing and statistical modeling were performed using IBM SPSS Statistics v25.0 (RRID:SCR_019096) and GraphPad Prism v9.0.0 (RRID:SCR_015807) with protocol-driven analytical pipelines. The statistical analyses were performed as follows: Gene expression correlations were assessed using Spearman's rank correlation analysis. Survival outcomes were evaluated by Kaplan–Meier survival curves with log-rank tests for comparative analysis. Differences between experimental groups were examined using Student's t-test for pairwise comparisons and one-way analysis of variance (ANOVA) for multiple group comparisons. A two-tailed *P* value < 0.05 was considered statistically significant throughout the study.

## Results

### SCs–GC crosstalk promotes PNI

**Fig. 1:**
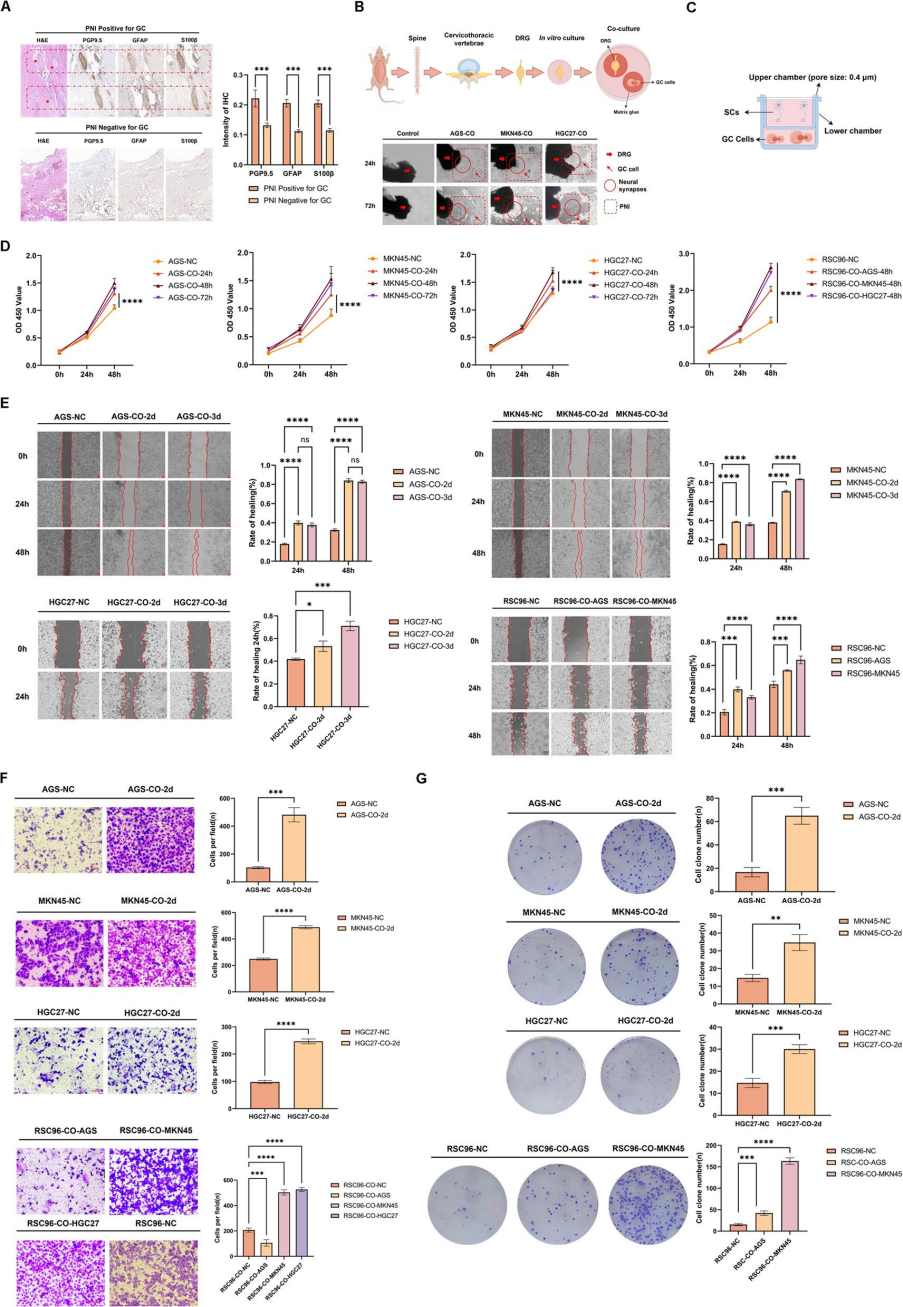
SCs–GC Interaction Drives PNI. **A** IHC identification of SCs in PNI-positive GC Tissues. **B** DRG-PNI co-culture model recapitulates PNI in Vitro**. C.** Schematic representation of an indirect co-culture model of SCs and GC cells. **D**-**G** Functional profiling of co-cultured cells: proliferation (CCk-8), migration (wound healing), invasion (transwell), and clonogenicity (colony formation)

To investigate the presence and functional role of SCs in GC, we analyzed 75 GC specimens from Gansu Provincial's Hospital, stratified into PNI-positive and PNI-negative groups based on pathological results. IHC staining for SCs markers (GFAP, S100β) and neural markers (PGP9.5) revealed that SCs and nerve fibers were significantly enriched in PNI-positive tumors (Fig. 1A). Combined with H&E staining, it is shown that the distribution of SCs and the regions where GC occurs PNI are highly overlapping and enriched.

The presence of SCs was found to be significantly associated with the occurrence of PNI. However, the question of whether there is any interaction between them remains unanswered. In order to verify the interaction between GC cells and nerves, a direct co-culture system of DRG and GC cells was established. The results (Fig. 1B) demonstrated that within the co-culture system, DRG facilitated the formation of new synapses over time, which dynamically extended in the direction of GC cells. The migration of GC cells along these DRG-derived synapses was observed, and it was noted that they broke through the matrix layer around the DRG. This process of migration was gradual, and it resulted in the wrapping of the DRG, thereby forming the PNI. Conversely, DRG neurons cultured in isolation exhibited no corresponding alterations with the passage of time and also demonstrated an absence of new synapse formation, suggesting that GC cells prompted the nerve tissue to generate synapses in the direction of the GC cells. Concurrently, the cells of GC migrate along the direction of nerve synapses and gradually form PNI. It can be concluded that there is a continuous and dynamic mutual communication between GC cells and neural tissue, which drives the formation of PNI in GC.

A robust interaction has been observed between GC cells and DRG. In the context of GC cells, DRG exhibits a propensity to generate a substantial number of synapses in the direction of GC cells. It has been reported that the process of neurogenesis at synapses is contingent on the presence of SCs [25, 26]. It is conceivable that the interaction between GC cells and DRG is facilitated by SCs. We proceeded to investigate the bidirectional interaction between GC and SCs using an indirect coculture system (Fig. 1C). CCK-8 results showed that GC cells co-cultured with SCs for 48 h showed the highest proliferation ability, and SCs also showed a significant increase in proliferation ability after co-cultured with GC cells. This observation was further substantiated by the wound healing assay, which demonstrated an increase in cell migration. Additionally, the Transwell assay revealed a significant increase in cell invasion, while the colony formation assay further corroborated these findings. These results, depicted in Fig. 1D, highlight the pronounced impact of the co-culture approach on the proliferation, migration, invasion, and colony formation of both GC and SCs when compared with cultures maintained in isolation. The findings demonstrate a mutually reinforcing relationship in which interactions between SCs and GC cells amplify their respective malignant phenotypes.

Collectively, our study identifies SCs as critical mediators in GC progression through neural crosstalk, providing mechanistic insights into neuro-invasive tumor behavior and potential therapeutic targets.

### SCs-induced PSAP overexpression potentiates GC malignancy

**Fig. 2:**
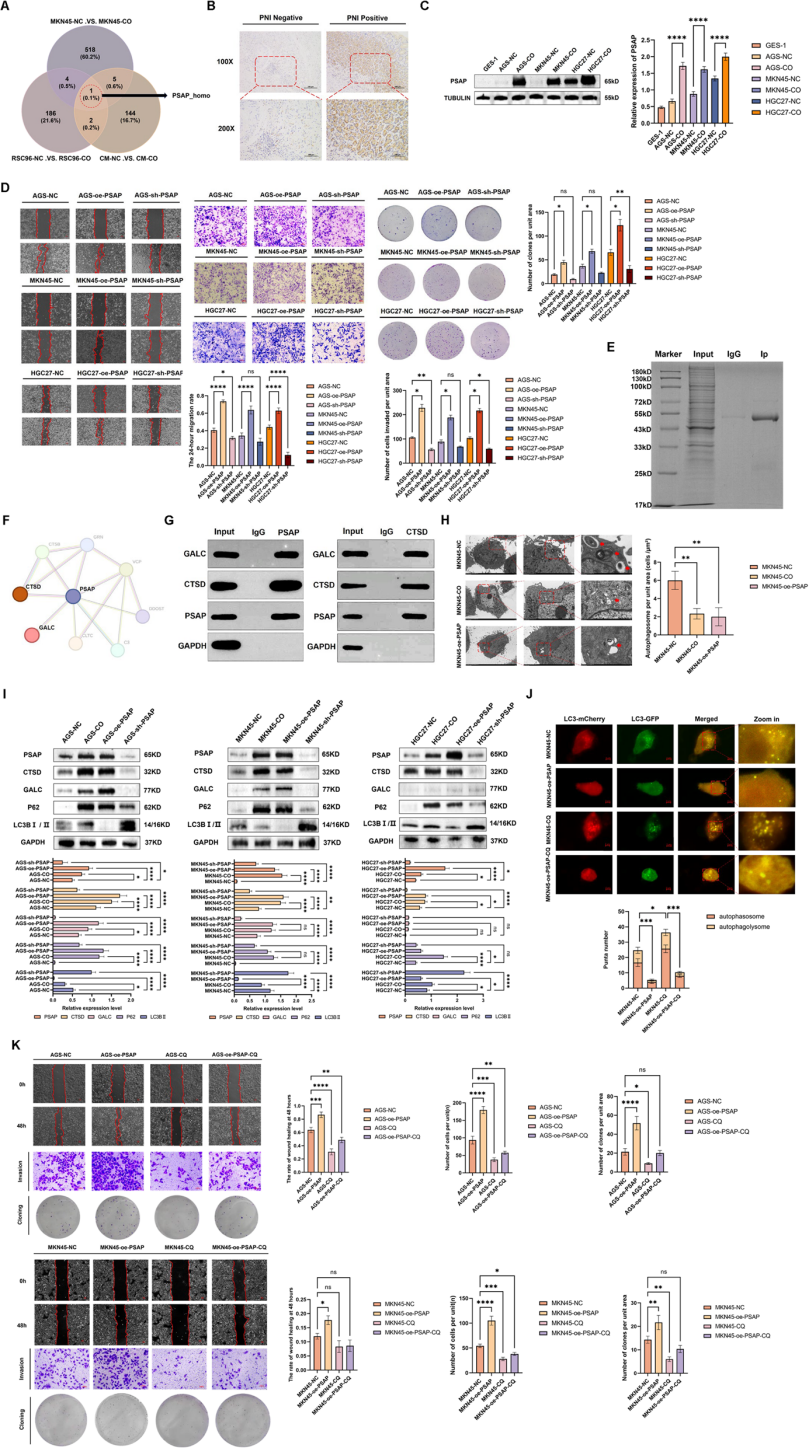
SCs-Induced PSAP Overexpression Potentiates GC Malignancy. **A** Venn analysis of differentially expressed proteins (DEPs) in the co-culture system. **B** IHC validation of PSAP upregulation in PNI-positive GC tissues. **C** Western Blot (WB) confirm PSAP Induction in co-cultured GC cells. **D** PSAP augments migration, invasion, and clonogenic capacity of GC cells. **E**–**F** The possible interacting proteins of PSAP were analyzed by co-immunoprecipitation and mass spectrometry. **G** Pull-down assay was used to verify the interaction of PSAP-CTSD-GALC. **H** Scanning electron microscopy was used to detect autophagosomes. **I** Inhibition of Autophagy Mediated by the PSAP–CTSD–GALC Complex. **J** mRFP-GFP-LC3B adenovirus reporter was used to detect autophagic flux. **K** Autophagic flux inhibition attenuates tumor-promoting effects of PSAP overexpression

To elucidate the underlying mechanisms of crosstalk between GC cells and SCs, we performed proteomic analysis of three components in their co-culture system: GC cells, SCs, and conditioned medium. Significantly differentially expressed proteins were screened for each fraction, as shown in (Supplementary Fig. 1B). We then performed Venn diagram analysis of the significantly differentially expressed proteins in each component, combined with species information, and further sorted out the significant changes in GC cells during this process. The results showed that human PSAP showed consistent up-regulation in all three components. This indicates the potential role of GC cell-derived PSAP in mediating GC-SC interaction (Fig. 2A).

In addition, IHC of PNI-positive and PNI-negative GC specimens confirmed that PSAP expression was significantly higher in PNI-positive tumors (*p* < 0.01, *n =* 75) (see Fig. 2B and Supplementary Fig. 1 C). In addition, the expression level of PSAP was significantly increased in both GC cells and SCs after co-culture (Figs. 2C). This finding confirmed the ability of SCs to induce high expression of PSAP in GC and that high expression of PSAP was significantly correlated with PNI status in GC.

PSAP was highly expressed in PNI-positive GC tissues and GC cells co-cultured with SCs. PSAP may play an important role in the interaction between SCs and GC cells and the occurrence and development of PNI in GC. Functionally, PSAP-OE GC cells exhibited significantly increased invasive, migratory and clonogenic capacities compared with control cells, whereas PSAP-KD cells displayed a markedly attenuated malignant phenotype (Fig. 2D). Based on Co-IP experiments and mass spectrometry analysis (Fig. 2E and Supplementary Fig. 1D), PSAP may interact with Granulin (GRN), galactocerebrosidase (GALC) and Cathepsin D (CTSD) to form complexes. It has been reported that the expression of PSAP and CTSD is impaired in response to specific light damage in colorectal cancer cells, leading to severe dysfunction of lysosomal function and autophagic flow, thereby inhibiting the development of colorectal cancer cells [27]. In addition, Ying Sun et al. [28] found that PSAP can be cleaved by CTSD to produce active Saposin, which activates GALC to participate in glycosphingolipid metabolism. However, Saposin deficiency leads to glycosphingolipid metabolism disorder, resulting in the accumulation of glycosphingolipid (especially galactosphingosine), leading to dysfunction of the lysosomal-autophagy system. These findings highlight a critical role for the PSAP–CTSD–GALC axis in maintaining lysosomal–autophagic homeostasis. Based on this rationale, we hypothesized that, under the influence of SCs, PSAP, CTSD and GALC assemble into a functional complex that perturbs lysosomal–autophagic regulation and thereby promotes PNI in GC.

Consistent with this hypothesis, pull-down assays and molecular docking simulations confirmed direct interactions among PSAP, CTSD and GALC (Fig. 2G and Supplementary Fig. 1E). Transmission electron microscopy (TEM) further revealed a reduced number of autophagosomes in GC cells co-cultured with SCs or PSAP-OE, compared with control cells (Fig. 2H). WB analysis showed increased CTSD and GALC expression accompanied by decreased autophagosome formation—as evidenced by p62 accumulation and a reduced LC3B-II/I ratio—in SCs co-cultured and PSAP-OE GC cells, whereas the opposite changes were observed in PSAP-KD GC cells (Fig. 2I). Using an mRFP–GFP–LC3B adenoviral reporter, PSAP-OE GC cells exhibited a marked increase in autophagic puncta after chloroquine (CQ, MCE, USA, #HY-17589A, 20μΜ, 24 h) treatment, indicating that autophagic flux remained functional and that PSAP primarily suppresses autophagosome formation rather than blocking autophagosome degradation (Fig. 2J). Notably, CQ treatment significantly attenuated the pro-malignant effects driven by PSAP-OE (Fig. 2K), further supporting a functional link between PSAP-dependent lysosomal–autophagic modulation and GC cell aggressiveness. Collectively, these data suggest that, in the context of SCs-mediated cues, PSAP promotes the assembly of a PSAP–CTSD–GALC complex that suppresses autophagy initiation and thereby enhances the malignant progression of GC cells within the tumor–nerve microenvironment, potentially contributing to PNI.

### Bidirectional PSAP-TGFβ1 paracrine signaling sustains SCs–GC crosstalk

The study demonstrated mutual activation between SCs and GC using a transwell chamber indirect co-culture system. The results demonstrated that SCs were capable of inducing high expression of PSAP in GC cells and promoting the malignant progression of these cells. Concurrently, the proliferation, migration, invasion and cloning ability of SCs within the co-culture system were also significantly enhanced. This finding suggests that the interaction between SCs and GC cells is bidirectional and can be facilitated through indirect pathways.

**Fig. 3:**
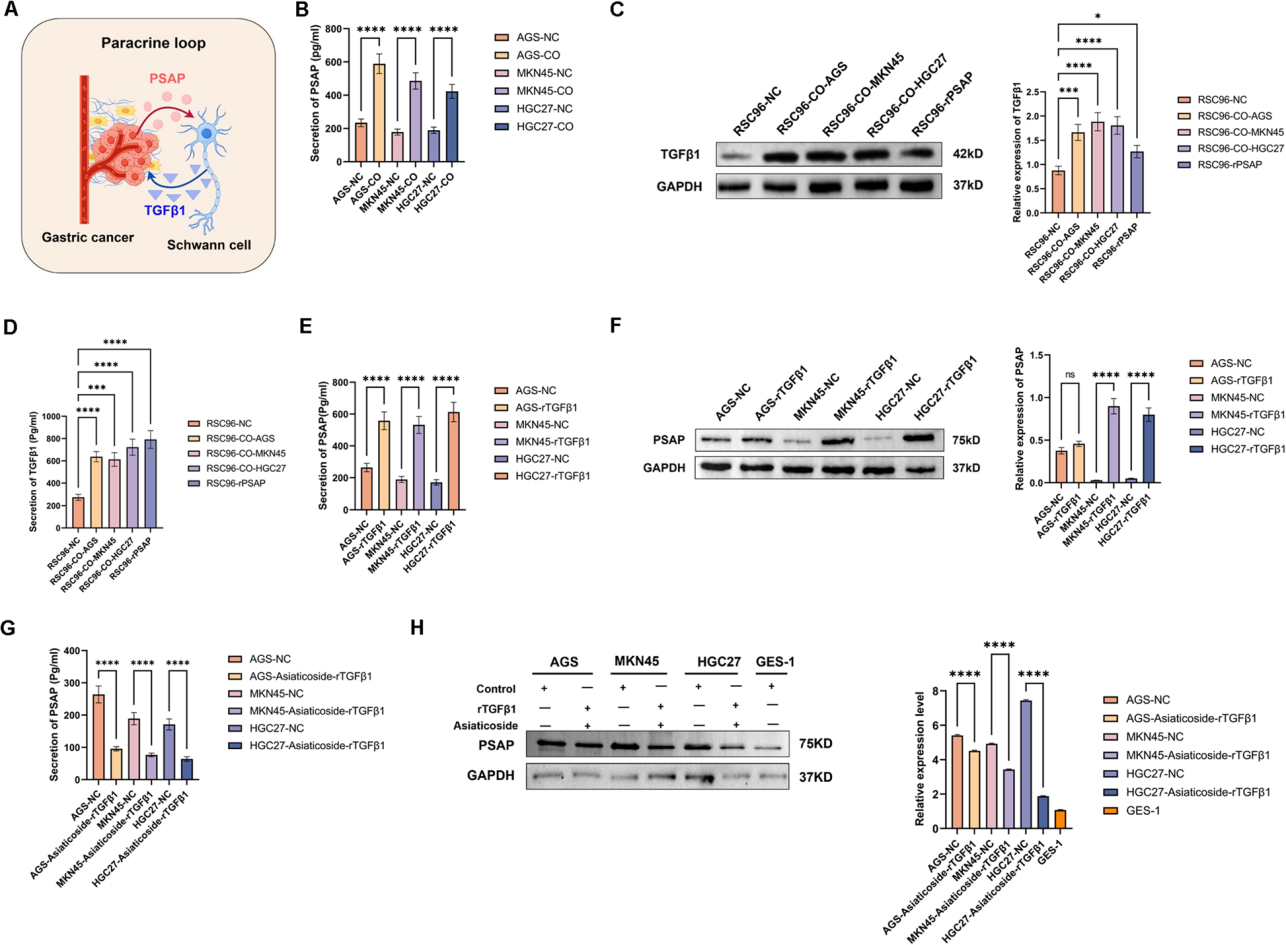
Bidirectional PSAP-TGFβ1 Paracrine Signaling Sustains SCs–GC Crosstalk. **A** Schematic of paracrine communication between SCs and GC Cells. **B** The secretion of PSAP by GC cells increased after co-culture with SCs. **C** TGFβ1 expression was increased in SCs co-cultured with GC cells and treated with rPSAP. **D** TGFβ1 secretion was increased in SCs co-cultured with GC cells and treated with rPSAP. **E**–**F** The secretion and expression of PSAP in GC cells treated with rTGFβ1 were increased. **G**-**H** The TGFβ1 inhibitor (Asiaticoside) can inhibit the effect of rTGFβ1 on the secretion and expression of PSAP

The paracrine pathway is considered the most classical and straightforward method to achieve indirect interaction between cells. Previously, we performed a comprehensive proteomic analysis of the three components in the co-culture system. It was found that in the co-culture system, GC cell-derived PSAP was highly enriched in culture medium and SCs, while SCs-derived TGFβ1 was also highly enriched in culture medium and GC. In addition, an enrichment of TGFβ1 derived from SCs was found in GC cells, and an enrichment of PSAP derived from GC cells was found in SCs. The results suggest that PSAP and TGFβ1 may be the key signalling nodes mediating the bidirectional interaction of GC-SCs (Fig. 3A). Furthermore, the co-culture of GC cells with SCs resulted in a significant increase in extracellular PSAP secretion, as detected by ELISA (Fig. 3B). Furthermore, both the expression and secretion of TGFβ1 were found to be significantly increased in SCs exposed to GC cells and recombinant PSAP (rPSAP) (MCE, USA, #HY-P76553, 50 ng/ml, 24 h) (Figs. 3C and D). Furthermore, the results demonstrate that the expression and secretion of PSAP in GC cells is promoted by recombinant TGFβ1(rTGFβ1) (MCE, USA, # HY-P7118,0.2 ng/ml,24 h) (Fig. 3E-F). However, this promotion is inhibited by the TGFβ receptor inhibitor, Asiaticoside (MCE, USA, #HY-N0439,100 μg/ml,24 h) (Fig. 3G-H). The data presented herein establish a novel feedforward signalling axis in which GC-derived PSAP stimulates TGFβ1 secretion by SCs, which in turn amplifies PSAP produced by GC cells (Fig. 3A). This self-perpetuating paracrine loop provides a mechanistic insight into the neurotropism of gastric malignancies, thus revealing potential therapeutic targets.

### PSAP activates GPR37/RAC1/ACTB signaling to drive TGFβ1 secretion in SCs

**Fig. 4:**
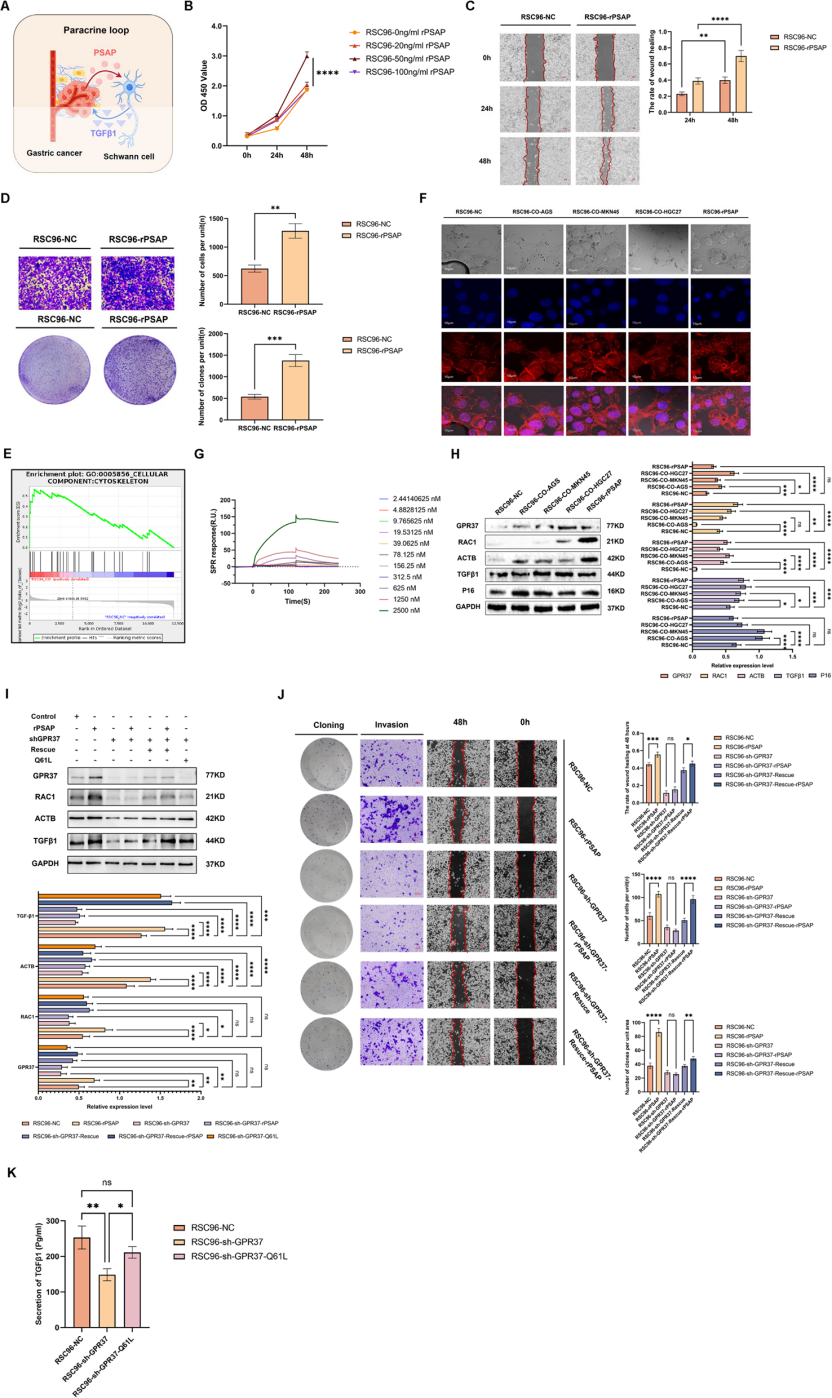
PSAP Activates GPR37/RAC1/ACTB Signaling to Drive TGFβ1 Secretion in SCs. **A** Schematic representation of PSAP secreted by GC cells acting on SCs. **B-D** rPSAP enhances SCs proliferation, migration, invasion and clonogenic capacity. **E** Functional enrichment analysis showed that the cytoskeleton of SCs changed after co-culture significantly. **F** Phalloidin staining reveals cytoskeletal changes in SCs following co-culture and rPSAP treatment. **G** SPR demonstrated direct binding between PASP and GPR37. **H** SCs co-cultured and exposed to rPSAP significantly activated GPR37/RAC1/ACTB signaling axis and TGFβ1 expression. **I-K** GPR37 sustains RAC1–ACTB signaling to drive TGF-β1 production and SC aggressiveness, conferring responsiveness to rPSAP

The interaction between GC cells and SCs is mediated by the PSAP-TGFβ1 signaling loop. GC-derived PSAP binds to SCs, reprograms the biological functions of SCs and promotes TGFβ1 secretion (Fig. 4A). To analyze the underlying mechanism, we treated SCs with rPSAP to mimic the effect of exogenous PSAP on SCs. The results of this experiment showed that rPSAP significantly increased the proliferation, migration, invasion, and colony formation abilities of SCs (Fig. 4B-D). Proteomic and functional enrichment analyses revealed significant changes in the cytoskeleton of SCs after co-culture with GC cells (Fig. 4E). Phalloidin staining showed that F-actin in SCs co-cultured and exposed to rPSAP formed typical stress fibers, which were arranged parallel to the long axis of the cells and enriched at the pseudopoda at the cell edge. The fluorescence intensity was enhanced, suggesting microfilament reorganization and cytoskeletal sclerosis (Fig. 4F).

Building on previous reports identifying GPR37 as a PSAP receptor [29–31], and that G protein-coupled receptors (GPCR) regulate axon growth, branching, and guidance through modulation of Rac1 GTP [32, 33], moreover, Rac1 GTP activation also regulates TGFβ1 expression [34, 35]. We hypothesized that PSAP signals through a GPR37–RAC1–actin axis in SCs. Using SPR, we confirmed a direct interaction between PSAP and GPR37 (Fig. 4G). Exposure of SCs to GC cells or rPSAP led to upregulation of GPR37, enhanced activation of the RAC1–ACTB pathway, increased expression of cytoskeleton-related markers, and a concordant rise in TGF-β1 levels (Fig. 4H). To define the functional relevance of GPR37, we generated SCs with stable GPR37-KD using lentiviral shGPR37. Silencing GPR37 attenuated RAC1–ACTB signaling, reduced TGF-β1 expression and secretion, and weakened the malignant phenotypes of SCs, including their proliferative, migratory, and invasive capacities; importantly, these effects were no longer responsive to rPSAP stimulation; rescue of GPR37 partially restored RAC1–ACTB activity, TGF-β1 production, and the associated aggressive behaviors (Fig. 4I–J). Moreover, in the context of low GPR37 expression, forced activation of RAC1 was sufficient to re-induce ACTB and to promote TGF-β1 secretion (Fig. 4I, K), placing RAC1 downstream of GPR37 and directly linking it to cytoskeletal remodeling and TGF-β1 regulation. Collectively, these data demonstrate that GC cell–derived PSAP engages GPR37 on SCs to activate RAC1–ACTB signaling, thereby driving cytoskeletal reorganization and axon guidance–like changes, enhancing TGF-β1 secretion, and ultimately amplifying paracrine pro-tumorigenic signaling within the GC–SCs niche.

### TGFβ1 engages the TGFβ1/Smad4/Sortilin axis to amplify PSAP secretion and PNI

**Fig. 5:**
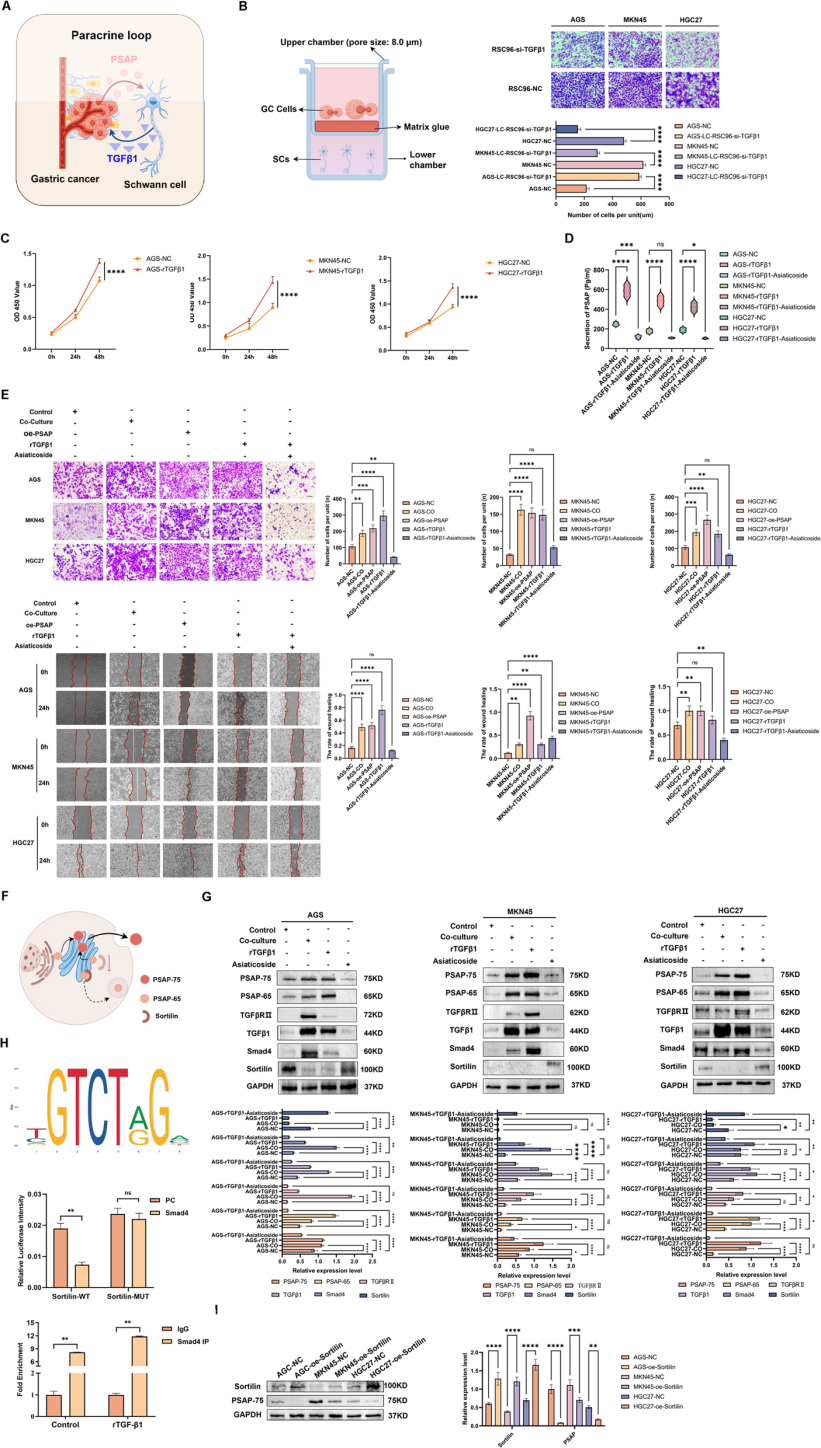
TGFβ1 Engages the TGFβ1/Smad4/Sortilin Axis to Amplify PSAP Secretion and PNI. **A** Schematic representation of TGFβ1 secreted by SCs acting on GC cells. **B** In *vitro* PNI assays showed that downregulation of SCs-derived TGF-β1 diminished GC cell PNI. **C-E** Asiaticoside can inhibit the effects of rTGFβ1 on the proliferation, invasion and PSAP secretion of GC cells. **F** Schematic representation of PSAP secretion. **G** TGFβ1/Smad4 signaling activation induces increased PSAP secretion through the suppression of Sortilin expression. **H** Dual-luciferase and ChIP-qPCR analyses showed that Smad4 binds the Sortilin promoter and represses its transcription, linking TGF-β1/Smad4 activation to Sortilin downregulation. **I** Sortilin overexpression suppresses PSAP secretion

PSAP secreted by GC cells binds to GPR37 on SCs and promotes the secretion of TGFβ1 by SCs. How does SCs-derived TGFβ1 promote PSAP secretion in GC cells (Fig. 5A)? To elucidate the molecular mechanisms underlying TGFβ1-driven malignant transformation and regulation of PSAP secretion, we first established an in *vitro* PNI model and demonstrated that SCs could induce PNI in GC cells, whereas this inductive capacity was markedly attenuated upon TGF-β1 downregulation (Fig. 5B and Supplementary Fig. 1 F). GC cells were treated with rTGFβ1 to mimic the effect of exogenous TGFβ1 on GC. The results of this assay showed that rTGFβ1 significantly promoted GC cell invasion, migration, and PSAP secretion (Fig. 5C-E). These effects were reproduced in co-culture of cells overexpressing PSAP and SCs and abolished by the TGFβR inhibitor Asaticoside (Fig. 5C-E). To analyze the regulation of PSAP secretion, we focused on its intracellular trafficking: PSAP-65(65KDa) is transported from the ER to the Golgi, where it is glycosylated to become PSAP-75(75KDa) and subsequently secreted. A fraction of PSAP-65 is retrogradely transported to lysosomes via Sortilin-mediated retrieval from the TGN for degradation [36–38]. Notably, TGF-β1-Sortilin interactions have been shown to modulate lysosomal degradation pathways [39, 40]. Based on the regulatory network of PSAP secretion, we hypothesized that TGF-β1 inhibits Sortilin expression, thereby blocking the delivery of PSAP-65 to lysosomes and enhancing extracellular secretion (Fig. 5F). WB confirmed that co-culture with SCs or exposure to rTGF-β1 led to decreased Sortilin expression and increased PSAP secretion in GC cells. Mechanistically, activation of the TGFβ1/Smad4 signaling pathway directly suppressed Sortilin expression (Fig. 5G). Dual-luciferase reporter assays combined with ChIP-qPCR further demonstrated that Smad4 directly binds to the Sortilin promoter and transcriptionally represses its activity, indicating that activation of the TGF-β1/Smad4 axis directly downregulates Sortilin (Fig. 5H). Conversely, enforced Sortilin-OE markedly decreased PSAP secretion (Fig. 5I), confirming a negative regulatory role of sortilin in PSAP trafficking.

Taken together, these findings support a model in which SCs-derived TGF-β1 engages TGF-βRII on GC cells to activate the TGF-β1/Smad4 pathway, leading to transcriptional repression of Sortilin, reduced lysosomal degradation of PSAP-65, and enhanced extracellular release of mature PSAP-75. This feed-forward mechanism strengthens GC–SCs interactions and accelerates PNI progression.

### Therapeutic targeting of PSAP/TGFβ1 suppresses PNI in vivo

**Fig. 6:**
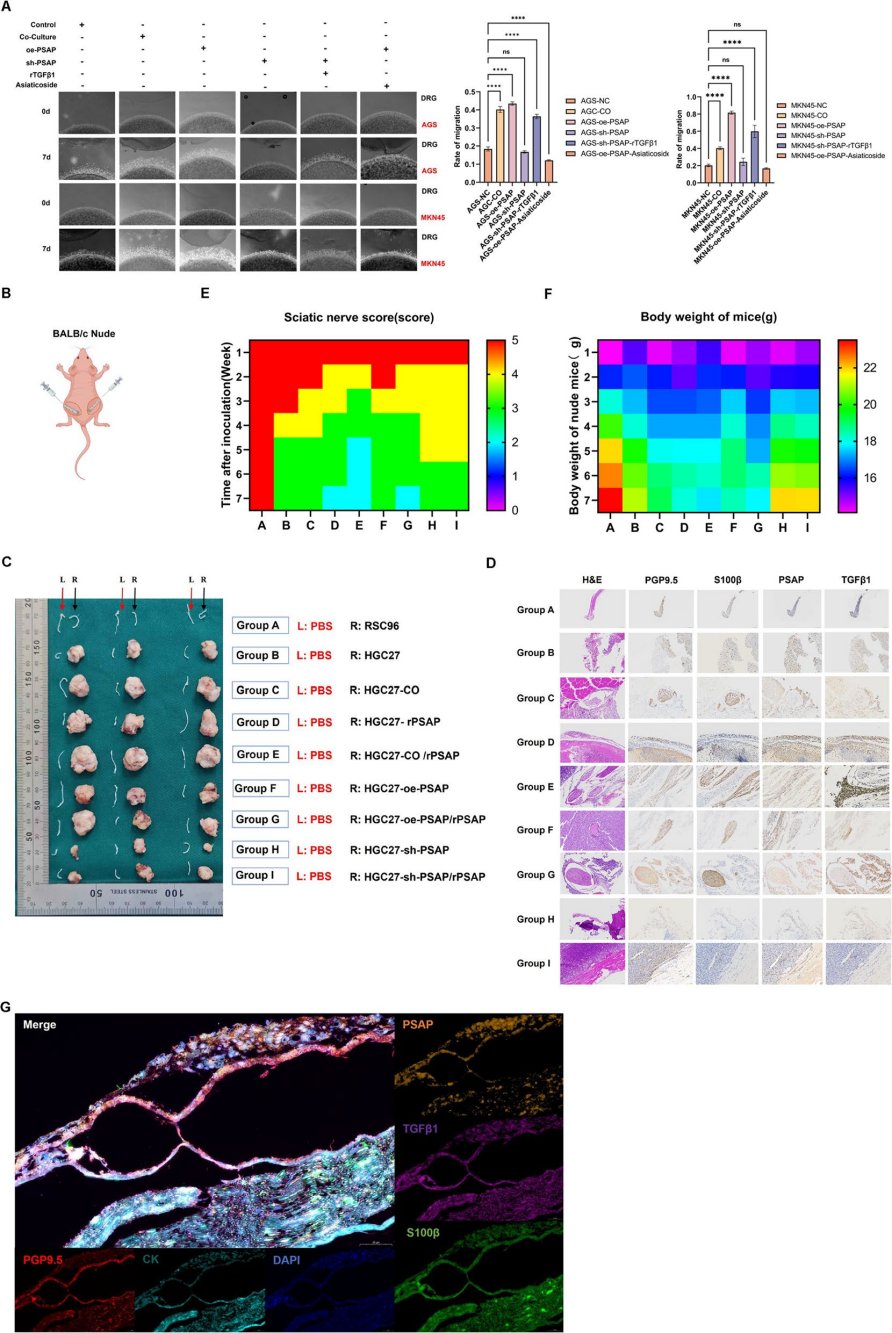
Therapeutic Targeting of PSAP/TGFβ1 Suppresses PNI In Vivo. **A** Inhibition of PSAP/TGFβ1 reduces PNI Incidence DRG-PNI model in vitro*.* **B** Schematic of a Sciatic Nerve Invasion Murine Model. **C-D** Tumor formation and H&E and IHC detection in the sciatic nerve model. **E**–**F** Functional and physiological assessments post intervention (Sciatic Nerve Score, Body Weight). **G** GMulticolor IF was used to verify the spatial distribution of PSAP, TGFβ1 and SCs(S100β) in PNI-positive tumor tissues

PSAP-TGFβ1 paracrine feedback loop connects SCs and GC, and plays an important role in the process of PNI in GC. By establishing matrigel/DRG co-culture system (Fig. 6A), SCs co-cultured GC cells showed significantly enhanced migration and invasion to DRG compared with the control group. GC cells OE PSAP reduplicated the SCs-induced PNI phenotype, while GC cells with PSAP KD showed reduced PNI ability. Addition of rTGFβ1 to PSAP KD GC cells partially restored the ability of PNI, and the TGFβR inhibitor Asiaticoside abolished the SCs-induced PNI enhancement.

In addition, a model of sciatic nerve invasion was established by injecting cell suspension into the right sciatic nerve of nude mice (left side: sterile PBS control) (Fig. 6B). The results of this experiment showed no tumor formation in PBS-injected mice (group A, left) and no tumorigenic potential in pure SCs (group A, right, Fig. 6C). Co-injection of GC cells with SCs showed a stronger tumor formation capacity than injection of GC cells alone (group B vs. group C, Fig. 6C). GC cells OE PSAP (group F) showed enhanced PNI compared to the control group (group C). Combined treatment of rPSAP and SCs produced a synergistic effect to promote PNI (group E vs. group D, Fig. 6C). PSAP KD GC cells exhibited reduced PNI, which was partially restored by the addition of rPSAP (group H-I, Fig. 6C). Both neurological function and physiology were greatly affected, with mice in groups D, E, and G exhibiting the most severe sciatic nerve dysfunction (Fig. 6E) and weight loss (Fig. 6F). H&E staining and IHC showed the most extensive PNI in groups D, E, and G (Fig. 6D and Supplementary Fig. 1E). S100β, PSAP and TGFβ1 were highly expressed in the PNI region of the tumor. mIHC confirmed the spatial colocalization of SCs, PSAP, and TGFβ1 in the PNI region (Fig. 6G). These data suggest an important role for PSAP-TGFβ1 in the process of tumor PNI and also further reveal potential therapeutic targets targeting tumor-nerve interactions.

### PSAP/TGFβ1/S100β multimodal signatures correlate with PNI and improve PNI discrimination

**Fig. 7:**
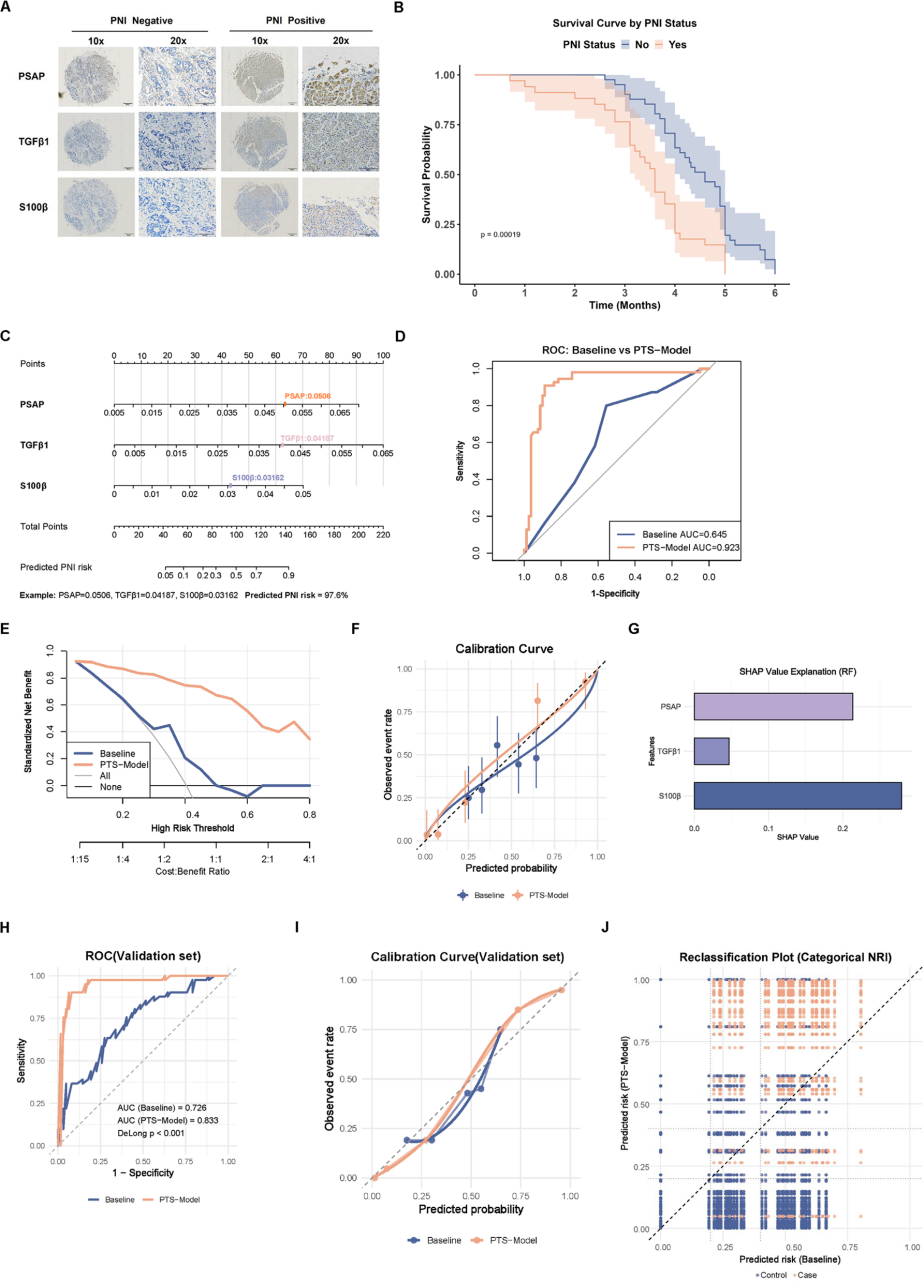
PSAP/TGFβ1/S100β multimodal signatures correlate with PNI and improve PNI discrimination. **A** IHC Staining of PSAP, TGFβ1, and S100β in PNI Subgroups. **B** Kaplan–Meier Survival Analysis by PNI Status in a Clinical Cohort. **C** Nomogram of PSAP, TGFβ1 and S100β combined to predict PNI. **D**-**F** ROC curve analysis, DCA, and calibration curves for comparison of PTS model and Baseline model for predicting PNI status. **G** The PTS model performance SHAP analysis. **H**-**I** ROC and calibration curves for the PTS and baseline models in an independent validation cohort. **J** Reclassification analysis of the PTS model and Baseline model

IHC staining was performed on tumor tissues from 75 patients. PNI-positive tumors exhibited significantly higher expression and cytoplasmic distribution of PSAP (median IHC score: 5.8 vs. 3.1, *p* < 0.001), TGF-β1 (median: 4.7 vs. 2.3, *p* < 0.001), and S100β (median: 6.2 vs. 3.4, *p* < 0.001) compared to PNI-negative tumors (Fig. 7A and Supplementary Fig. 2 A). A retrospective analysis was conducted on 75 gastric adenocarcinoma patients diagnosed at Gansu Provincial’s Hospital (January 2018–January 2019). Patients were stratified into PNI-positive (*n =* 34) and PNI-negative (*n =* 41) groups based on pathological reports. Kaplan–Meier analysis revealed significantly shorter overall survival in the PNI-positive group (*p* < 0.001, log-rank test; Fig. 7B). Quantitative analysis demonstrated strong positive correlations between biomarkers (PSAP-TGF-β1: *r =* 0.55, *p* < 0.001; TGF-β1-S100β: *r =* 0.82, *p* < 0.001; PSAP-S100β: *r =* 0.47, *p* < 0.001; Supplementary Fig. 2B). Multivariate logistic regression analysis revealed strong associations between PNI status and expression levels of PSAP ((odds ratis(OR) = 4.32, 95% confidence interval (CI) = 2.15–8.67, *p* < 0.001), TGFβ1 (O*R =* 3.89, 95% CI = 1.98–7.64, *p* = 0.002), and S100β (O*R =* 5.11, 95% CI = 2.45–10.65, *p* < 0.001) in GC tissues (Supplementary Fig. 2 C).

In order to obtain a clearer readout of PNI, a PTS model was built using a machine-learning approach informed by the expression levels of PSAP, TGF-β1, and S100β. This model is presented as a scatter plot (Fig. 7C). In the course of the experimental procedure, the random forest classifier yielded a reasonably accurate separation of PNI categories, attaining an AUC (area under the curve) = 0.923(95%CI 0.846 −1.000). Conversely, a model that relied exclusively on conventional clinical descriptors—namely, TNM classification, lymph node involvement, and vascular invasion—yielded an considerably lower AUC = 0.645(95%CI 0.591–0.787) (Fig. 7D). A comprehensive evaluation of the PTS model reveals its superior capacity to detect molecular signals associated with PNI when compared to conventional clinical staging methods. The clinical decision curve analysis (DCA) and calibration curve and showed that the prediction sensitivity, accuracy and clinical application value of the prediction model had excellent performance (Fig. 7E-F). Feature importance analysis indicated that S100β and PSAP contributed 3.2-fold higher predictive weight than TGF-β1 (mean decrease in Gini index: S100β = 0.38, PSAP = 0.29, TGF-β1 = 0.11; Fig. 7G). The high performance of PSAP/TGFβ1/S100β combination in predicting PNI further indicates the potential research and clinical application value of PSAP/TGFβ1/S100β in the future treatment of GC.

In order to enhance the robustness and generalisability of the model, an external validation cohort was also established, which included 93 patients with GC. An independent dual-center dataset was formed by the collection of clinical and pathological variables, with geographical differences ensuring strict separation of the training and validation samples, thereby minimising the risk of data leakage to the greatest extent. The performance of the PTS model and the clinical baseline model was evaluated using the external validation cohort. ROC analysis revealed that the PTS model exhibited strong discriminatory capacity in the independent dataset, with an AUC = 0.833,95%CI 0.71—0.903. This performance surpassed that of the baseline model, as depicted in Fig. 7H. This finding is consistent with the pre-defined calibration assessment, which indicated that the calibration slope of the PTS model was closer to 1 and the intercept was closer to 0, meaning that the consistency between the predicted risk and the actual risk was better than the baseline method (Fig. 7I). The reclassification analysis further demonstrated that the risk stratification provided by the PTS model had a substantial improvement. The unclassified NRI (Net Reclassification Improvement) was 1.379 (95% CI 1.132—1.634), indicating that, in comparison with the baseline clinical model, this model accurately reallocated the majority of patients to more suitable risk categories (Fig. 7J). Additionally, the IDI (Integrated Discrimination Improvement) was 0.472 (95% CI 0.393—0.550), reflecting a significant improvement in the average sensitivity of the prediction, but without a corresponding loss in specificity. These findings confirmed that the PTS model provided significant incremental value than baseline model.

These findings support that PSAP/TGF-β1/S100β-associated multimodal signatures are significantly correlated with PNI incidence in GC patients and provide improved discriminative and risk-stratification utility beyond conventional clinicopathological factors, suggesting their potential as a complementary tool for PNI risk assessment.

## Discussion

Distant metastasis and directional migration of tumor cells to neural pathways are one of the key factors determining tumor invasiveness and prognosis. In addition to the traditional lymphatic and hematogenous metastasis, PNI is also a metastasis pattern with independent clinical significance [41–44]. A mounting body of evidence suggests that PNI is not merely a process in which tumor cells "passively crawl" along nerves, but rather an active, highly orchestrated event driven by multiple paracrine signaling pathways between tumor cells and nerves/glial cells. However, in the context of GC, the systematic elucidation of how tumor cells engage specific molecular networks to interact with the peripheral nervous system and drive PNI remains to be elucidated.

Against this background, the main findings of the present study can be summarized at three levels: (1) At the mechanistic level, we identify tumor cell–derived PSAP as a key factor mediating PNI-associated neural remodeling in GC; (2) At the signaling network level, we delineate a feedforward–feedback loop composed of PSAP–GPR37–RAC1–TGFβ1–Sortilin, which functionally links intracellular autophagy inhibition in tumor cells to paracrine crosstalk between tumor cells and SCs; and (3) At the translational level, we show that combined expression of PSAP/TGFβ1/S100β can be used to construct a PNI prediction model with high discriminative performance in our cohort, suggesting its potential diagnostic and prognostic value.

In our in *vitro* experiments, we therefore focused on SCs, which are critical regulators of peripheral nerve function and have been implicated as active participants in tumor initiation and progression through paracrine signaling [45, 46], although the initiating signals that recruit them into pro-tumorigenic roles remain elusive. First, the activation effect of PSAP on SCs was verified, and then previous studies have identified PSAP-GPR37 axis as a key node in neuron-glial communication in the central nervous system: PSAP-GPR37 signaling aggravated neuroinflammation and promoted neurodegeneration by inducing IL-6 secretion from oligodendrocytes in a PD model; However, rPSAP exerts neuroprotective and vascular protective effects through the GPR37/PI3K/Akt/ASK1 pathway in the ischemic brain injury model [29, 30, 47–49]. Based on this knowledge, our study extends the biological scope of PSAP-GPR37 axis from "damaged central nervous system" to "tumor-peripheral nerve interface" and reveals its novel function in the PNI microenvironment of GC.

We found that SCs intrinsically express GPR37, and its expression is markedly upregulated when SCs are co-cultured with GC cells or exposed to rPSAP. Proteomic functional enrichment analysis showed that “cytoskeletal remodeling” is one of the most prominently altered biological processes in SCs after co-culture with GC cells. Phalloidin staining further confirmed that co-culture or PSAP stimulation induces prominent accumulation of stress fibers and reorganization of actin filaments in SCs (Fig. 4F). This pattern closely mirrors the RAC1-dependent cytoskeletal rearrangements that occur during peripheral nerve development, when SCs extend processes and intercalate into axon bundles to accomplish radial sorting and subsequent myelination [50–52]. Together with the report by Ungfroren et al. that RAC1 promotes autocrine TGFβ1 production [34], our data support a signaling axis whereby PSAP activates RAC1 in SCs via GPR37, thereby driving cytoskeletal remodeling and enhancing TGFβ1 secretion. This establishes a PSAP/GPR37–RAC1/ACTB–TGFβ1 paracrine axis between GC cells and SCs. Functionally, this axis promotes directional migration of glial cells toward nerve ganglia on the one hand, and on the other, provides a persistent pro-invasive input that facilitates PNI.

Notably, this study not only elucidates the role of PSAP in tumor–nerve crosstalk but also clarifies its pro-tumorigenic mechanisms within tumor cells themselves. Previous literature has indicated that PSAP promotes tumor cell proliferation and survival by activating classical signaling pathways such as MAPK, PI3K/Akt–GSK3α/β, and TGFβ1, and reprograms tumor energy metabolism by facilitating coenzyme Q10 binding and transport [53]. Aberrant glycosylation mediated by PSAP has also been implicated in remodeling the immune microenvironment and modulating responses to immunotherapy [36, 54]. Consistent with these observations, we show that PSAP overexpression significantly enhances GC cell migration, invasion, and clonogenicity, whereas PSAP knockout markedly suppresses these malignant phenotypes (Fig. 2I), indicating a typical oncogenic role of PSAP in GC.

To further dissect the intracellular function of PSAP, we identified a PSAP/CTSD/GALC complex by co-immunoprecipitation coupled with mass spectrometry and subsequently confirmed the existence of this complex using pull-down assays. Altered levels and enzymatic activity of CTSD can disrupt lysosomal homeostasis and impair autophagosome degradation [27, 55, 56], while dysregulation of GALC perturbs sphingolipid metabolism and affects PSAP degradation, leading to accumulation of toxic metabolites and blockage of autophagic flux [28]. Our results extend these findings by demonstrating that the PSAP/CTSD/GALC complex directly suppresses autophagic activity and promotes malignant behavior of GC cells. By manipulating PSAP expression, we indirectly modulate levels of this complex and observe concordant changes in tumor cell migration, invasion, and clonogenic capacity. These data suggest that PSAP maintains a more invasive and therapy-tolerant tumor cell state at the intracellular level by stabilizing the PSAP/CTSD/GALC complex and inhibiting autophagy.

Concurrently, our study reveals a “signal amplification linkage” between extracellular PSAP and the TGFβ1/Smad–Sortilin pathway. Extracellular PSAP activates SCs to secrete TGFβ1, which in turn acts on GC cells via the TGFβ1–Smad pathway to downregulate Sortilin, thereby reducing lysosomal targeting of PSAP and further enhancing its secretion. Previous studies have suggested that excessive glycosylation can promote PSAP secretion [36]. Our findings indicate that TGFβ1/Smad signaling represents another crucial upstream regulator of PSAP release. Considering the established role of TGFβ1 in promoting epithelial–mesenchymal transition (EMT) and invasion in GC [57–59], our data place PSAP upstream of TGFβ1 signaling and integrate autophagy inhibition, glial activation, and paracrine positive feedback into a unified mechanistic framework.

Conceptually, our results organize PNI-related PSAP biology into three interlocked functional modules: (1) an intracellular PSAP/CTSD/GALC module, which disrupts lysosome–autophagy homeostasis and maintains a highly malignant tumor cell state; (2) a tumor–glial paracrine module, in which tumor-derived PSAP drives glial activation and nerve-directed migration via the GPR37–RAC1–TGFβ1 axis, thereby providing essential microenvironmental support for PNI; and (3) a TGFβ1/Smad–Sortilin amplification module, which suppresses Sortilin, enhances PSAP secretion, and thereby strengthens the signaling intensity of the first two modules. Together, these three modules form a feedforward–feedback network that offers a concise and systematic framework for understanding signal integration in GC–associated PNI.

At the level of cellular behavior, our study supports the view that PNI should be regarded as a “cooperative invasion” process jointly executed by tumor cells and glial cells, rather than a unidirectional invasion by a single cell population. Our data show that GC cells possess an intrinsic propensity for neural invasion, while in the presence of tumor cells, SCs display enhanced migratory capacity and a remodeled secretory profile that actively shapes a local microenvironment permissive to PNI. This leads to a conceptual model in which tumor cells and nerve/stromal cells form a dynamic, mutually reinforcing network that collectively drives the initiation and progression of PNI [60]. It is important to emphasize that such a “cooperative mode” is likely to exhibit temporal and spatial heterogeneity, with the relative contribution of different cellular populations varying across tumor subtypes and stages of invasion. Future studies combining in vivo real-time imaging at the invasive front with cell type–specific conditional genetic manipulations will be instrumental for dissecting the hierarchical roles of distinct cell populations in PNI at single-cell and spatiotemporal resolutions.

Clinically, PSAP and TGFβ1 have been reported as prognosis-related biomarkers in several malignancies, including glioblastoma and pancreatic cancer [61–63]. In our GC cohort, the levels of PSAP, TGFβ1, and S100β were significantly higher in PNI-positive patients than in PNI-negative patients. Based on these findings, we used machine learning methods to construct a PTS predictive model integrating the expression of these three markers. This model achieved an accuracy over 80% in identifying PNI, and combined expression of PSAP/TGFβ1/S100β was significantly associated with PNI status (Fig. 7A–C). It should be noted, however, that our study is limited by a relatively small sample size and the single-center, retrospective design. An independent external validation cohort was assembled from two geographically distinct branch hospitals within the same institutional network, and the classifier was evaluated using a locked model (no retraining or recalibration) with no overlap between derivation and validation cohorts. This provides stronger evidence of transportability than internal resampling alone; however, because validation was conducted within a single healthcare system, broader generalizability will require confirmation in truly multi-institutional cohorts across independent systems. At present, therefore, the PTS model is best regarded as a “candidate biomarker panel,” and its true clinical utility will require confirmation in larger, multicenter, prospective cohorts.

Despite providing a relatively systematic depiction of the PSAP–TGFβ1 axis in GC PNI at mechanistic, functional, and clinical levels, several key questions remain unresolved. First, our current understanding of this feedforward–feedback circuit is largely derived from endpoint measurements and static omics data, which are insufficient to precisely delineate its temporal dynamics and the initial triggering events. Future studies could incorporate time-resolved live-cell imaging (such as rapid Ca^2^⁺ or ERK activity reporters) and compartmentalized/microfluidic co-culture systems featuring unidirectional signaling to identify the earliest responding cell populations and the directionality of signal propagation [64, 65]. Second, although cross-species co-culture and validation in clinical samples have increased the translational relevance of our findings to some extent, our current models still fall short of fully recapitulating the complexity of the human peripheral nervous system. Further validation in humanized nervous system models will be a critical step toward clinical translation. Third, the in *vivo* and ex *vivo* PNI models employed (DRG co-culture and sciatic nerve assays) are experimentally tractable and widely used for tumor–nerve interaction studies, but they do not fully recapitulate the gastric niche; orthotopic GC models incorporating quantitative PNI endpoints and cell type–resolved temporal profiling will further strengthen physiological inference. Fourth, given the high complexity of the tumor microenvironment (TME), this study primarily focuses on the PSAP/TGFβ1 axis between GC cells and SCs, without systematically evaluating the regulatory and feedback influences of fibroblasts, immune cells, endothelial cells, and extracellular vesicles on this axis. Finally, the PTS model requires validation and prospective evaluation in larger, truly multi-institutional cohorts and across treatment contexts will be required to define generalizability and clinical utility more rigorously, and its predictive performance across different populations and therapeutic contexts remains to be fully determined.

In summary, this study systematically identifies a PSAP–TGFβ1-centered feedforward–feedback signaling circuit in GC that integrates intrinsic autophagy inhibition within tumor cells, paracrine crosstalk between tumor cells and SCs, and the expression of clinically relevant biomarkers, thereby playing a critical driving role in the formation of PNI. By targeting key nodes within this circuit, we demonstrate that PSAP is essential for maintaining PNI and promoting malignant behavior of GC cells, and we provide multi-layered evidence supporting PSAP as a potential diagnostic/prognostic biomarker and therapeutic target. Looking ahead, as our understanding of the temporal dynamics of this circuit, the involvement of additional TME components, and the results of clinical cohort validation are further refined, PSAP and its downstream axis may form the basis for stratified diagnostic strategies and precision interventions, offering new tools for risk stratification and therapeutic targeting in patients with PNI-associated GC.

## Conclusion

This study delineates a pivotal mechanism whereby bidirectional crosstalk between GC cells and SCs drives PNI. We demonstrate that a PSAP–TGFβ1 axis operates as a self-reinforcing paracrine positive-feedback loop between GC cells and SCs, remodeling the tumor–nerve microenvironment, intensifying tumor–nerve interactions, and thereby promoting PNI progression. Collectively, these findings establish a mechanistic framework for PNI in GC and suggest that therapeutic disruption of the PSAP–TGFβ1 circuit and its feedback amplification may hold translational potential.

## Supplementary Information

Supplementary Material 1.

Supplementary Material 2.

## Data Availability

The data presented in this study are available through the corresponding author on reasonable request.

## Abbreviations

PNI: Perineural invasion PNI
GC: Gastric cancer
SCs: Schwann cells
PSAP: Prosaposin
CTSD: Cathepsin D
GALC: Galactocerebrosidase
GPR37: G protein–coupled receptor 37
TGFβ1: Transforming growth factor-β-1
TGFβRII: Transforming growth factor-β receptor-II
ER: Endoplasmic reticulum
TGN: Trans-Golgi network
FBS: Fetal bovine serum
P/S: Penicillin/streptomycin
DMSO: Dimethyl sulfoxide
CM: Conditioned media
DIA: Data-independent acquisition
SPR: Surface Plasmon Resonance
ChIP: Chromatin Immunoprecipitation
OE: Overexpression
NC: Non-promoter construct
Si: Small interfering
sh: Short hairpin
KD: Knockdown
CCK8: Cell Counting Kit-8
DRG: Dorsal Root Ganglion
WB: Western Boltting
IHC: Immunohistochemistry
FFPE: Formalin-fixed paraffin-embedded
mIHC: Multiplex Immunofluorescence Staining
TSA: Tyramide signal amplification
ANOVA: One-way analysis of variance
TEM: Transmission electron microscopy
rPSAP: Recombinant PSAP
rTGFβ1: Recombinant TGFβ1
GPCR: G protein-coupled receptors
OR: Odds ratis
AUC: Area under the curve
CI: Confidence interval
DCA: Decision curve analysis
NRI: Net Reclassification Improvement
IDI: Integrated Discrimination Improvement
EMT: Epithelial–mesenchymal transition
TME: Tumor microenvironment

## References

[1] Sung H, Ferlay J, Siegel RL, Laversanne M, Soerjomataram I, Jemal A, et al. Global cancer statistics 2020: GLOBOCAN estimates of incidence and mortality worldwide for 36 cancers in 185 countries. CA Cancer J Clin. 2021;71(3):209–49. 33538338 10.3322/caac.21660

[2] Han B, Zheng R, Zeng H, Wang S, Sun K, Chen R, et al. Cancer incidence and mortality in China, 2022. J Natl Cancer Cent. 2024;4(1):47–53. 39036382 10.1016/j.jncc.2024.01.006 PMC11256708

[3] Joshi SS, Badgwell BD. Current treatment and recent progress in gastric cancer. CA Cancer J Clin. 2021;71(3):264–79. 33592120 10.3322/caac.21657 PMC9927927

[4] Wang K, Zhao XH, Liu J, Zhang R, Li JP. Nervous system and gastric cancer. Biochim Biophys Acta Rev Cancer. 2020;1873(1):188313. 31647986 10.1016/j.bbcan.2019.188313

[5] Ajani JA, D’Amico TA, Bentrem DJ, Chao J, Cooke D, Corvera C, et al. Gastric Cancer, Version 2.2022, NCCN Clinical Practice Guidelines in Oncology. J Natl Compr Canc Netw. 2022;20(2):167–92. 35130500 10.6004/jnccn.2022.0008

[6] Benson AB, Venook AP, Al-Hawary MM, Arain MA, Chen YJ, Ciombor KK, et al. Colon cancer, Version 2.2021, NCCN clinical practice guidelines in oncology. J Natl Compr Canc Netw. 2021;19(3):329–59. 33724754 10.6004/jnccn.2021.0012

[7] Jiang N, Deng JY, Liu Y, Ke B, Liu HG, Liang H. Incorporation of perineural invasion of gastric carcinoma into the 7th edition tumor-node-metastasis staging system. Tumour Biol. 2014;35(9):9429–36. 24972970 10.1007/s13277-014-2258-5

[8] Bilici A, Seker M, Ustaalioglu BB, Kefeli U, Yildirim E, Yavuzer D, et al. Prognostic significance of perineural invasion in patients with gastric cancer who underwent curative resection. Ann Surg Oncol. 2010;17(8):2037–44. 20333555 10.1245/s10434-010-1027-y

[9] Lyu Y, Xie F, Chen B, Shin WS, Chen W, He Y, et al. The nerve cells in gastrointestinal cancers: from molecular mechanisms to clinical intervention. Oncogene. 2024;43(2):77–91. 38081962 10.1038/s41388-023-02909-x PMC10774121

[10] Li N, Tong H, Hou W, Liu Q, Xiang F, Zhu JW, et al. Neural-cancer crosstalk: reciprocal molecular circuits driving gastric tumorigenesis and emerging therapeutic opportunities. Cancer Lett. 2025;616:217589. 40015663 10.1016/j.canlet.2025.217589

[11] Zhao CM, Hayakawa Y, Kodama Y, Muthupalani S, Westphalen CB, Andersen GT, et al. Denervation suppresses gastric tumorigenesis. Sci Transl Med. 2014;6(250):250ra115. 25143365 10.1126/scitranslmed.3009569 PMC4374618

[12] Villanueva MT. Therapeutics: Gastric cancer gets a red carpet treatment. Nat Rev Cancer. 2014;14(10). 10.1038/nrc3825 25230885

[13] Vagal innervation is necessary for gastric tumorigenesis. Cancer Discov. 2014;4(11):Of15. 10.1158/2159-8290.CD-RW2014-187 25367956

[14] Amit M, Na’ara S, Gil Z. Mechanisms of cancer dissemination along nerves. Nat Rev Cancer. 2016;16(6):399–408. 27150016 10.1038/nrc.2016.38

[15] Liebig C, Ayala G, Wilks JA, Berger DH, Albo D. Perineural invasion in cancer: a review of the literature. Cancer. 2009;115(15):3379–91. 19484787 10.1002/cncr.24396

[16] Liu Q, Ma Z, Cao Q, Zhao H, Guo Y, Liu T, et al. Perineural invasion-associated biomarkers for tumor development. Biomed Pharmacother. 2022;155:113691. 36095958 10.1016/j.biopha.2022.113691

[17] He K, Wang H, Huo R, Jiang SH, Xue J. Schwann cells and enteric glial cells: emerging stars in colorectal cancer. Biochim Biophys Acta Rev Cancer. 2024;1879(5):189160. 39059672 10.1016/j.bbcan.2024.189160

[18] Cai Z, Yao H, Chen J, Ahmed AA, Li C, Hu X, et al. Schwann cells in pancreatic cancer: unraveling their multifaceted roles in tumorigenesis and neural interactions. Cancer Lett. 2024;587:216689. 38367898 10.1016/j.canlet.2024.216689

[19] Deborde S, Wong RJ. The role of Schwann cells in cancer. Adv Biol (Weinh). 2022;6(9):e2200089. 35666078 10.1002/adbi.202200089 PMC9474572

[20] Gao X, Wang Q, Huang T, Xu C, Yang X, Zhang L, et al. Cervical cancer-produced neuromedin-B reprograms Schwann cells to initiate perineural invasion. Cell Death Dis. 2024;15(8):636. 39214988 10.1038/s41419-024-07030-9 PMC11364772

[21] Grada A, Otero-Vinas M, Prieto-Castrillo F, Obagi Z, Falanga V. Research techniques made simple: analysis of collective cell migration using the wound healing assay. J Invest Dermatol. 2017;137(2):e11–6. 28110712 10.1016/j.jid.2016.11.020

[22] Gil Z, Rein A, Brader P, Li S, Shah JP, Fong Y, et al. Nerve-sparing therapy with oncolytic herpes virus for cancers with neural invasion. Clin Cancer Res. 2007;13(21):6479–85. 17975160 10.1158/1078-0432.CCR-07-1639

[23] Gil Z, Cavel O, Kelly K, Brader P, Rein A, Gao SP, et al. Paracrine regulation of pancreatic cancer cell invasion by peripheral nerves. J Natl Cancer Inst. 2010;102(2):107–18. 20068194 10.1093/jnci/djp456 PMC2911041

[24] Ayala GE, Wheeler TM, Shine HD, Schmelz M, Frolov A, Chakraborty S, et al. In vitro dorsal root ganglia and human prostate cell line interaction: redefining perineural invasion in prostate cancer. Prostate. 2001;49(3):213–23. 11746267 10.1002/pros.1137

[25] Gould TW, Ko CP, Willison H, Robitaille R. Perisynaptic Schwann cells: guardians of neuromuscular junction integrity and function in health and disease. Cold Spring Harb Perspect Biol. 2025;17(1):a041362. 10.1101/cshperspect.a041362 PMC11694759 38858074

[26] Sardella-Silva G, Mietto BS, Ribeiro-Resende VT. Four seasons for Schwann cell biology, revisiting key periods: development, homeostasis, repair, and aging. Biomolecules. 2021;11(12):1887. 10.3390/biom11121887 PMC8699407 34944531

[27] Yaya-Candela AP, Ravagnani FG, Dietrich N, Sousa R, Baptista MS. Specific photodamage on HT-29 cancer cells leads to endolysosomal failure and autophagy blockage by cathepsin depletion. J Photochem Photobiol B. 2024;255:112919. 38677261 10.1016/j.jphotobiol.2024.112919

[28] Sun Y, Grabowski GA. Altered autophagy in the mice with a deficiency of saposin A and saposin B. Autophagy. 2013;9(7):1115–6. 23697974 10.4161/auto.24919 PMC3722325

[29] Ma Q, Tian JL, Lou Y, Guo R, Ma XR, Wu JB, et al. Oligodendrocytes drive neuroinflammation and neurodegeneration in Parkinson’s disease via the prosaposin-GPR37-IL-6 axis. Cell Rep. 2025;44(2):115266. 39913287 10.1016/j.celrep.2025.115266

[30] Yu J, Li J, Matei N, Wang W, Tang L, Pang J, et al. Intranasal administration of recombinant prosaposin attenuates neuronal apoptosis through GPR37/PI3K/Akt/ASK1 pathway in MCAO rats. Exp Neurol. 2024;373:114656. 38114054 10.1016/j.expneurol.2023.114656 PMC10922973

[31] Meyer RC, Giddens MM, Schaefer SA, Hall RA. GPR37 and GPR37L1 are receptors for the neuroprotective and glioprotective factors prosaptide and prosaposin. Proc Natl Acad Sci U S A. 2013;110(23):9529–34. 23690594 10.1073/pnas.1219004110 PMC3677493

[32] Yang X, Pan C, Ye M, Liang J, Cheng H, Liang Q, et al. *Drosophila* adhesion GPCR remoulade regulates axon growth, branching, and guidance by modulating Rac1 GTPase. J Genet Genomics. 2024;51(4):458–61. 38049063 10.1016/j.jgg.2023.11.006

[33] Ng J, Nardine T, Harms M, Tzu J, Goldstein A, Sun Y, et al. Rac GTPases control axon growth, guidance and branching. Nature. 2002;416(6879):442–7. 11919635 10.1038/416442a

[34] Ungefroren H, Otterbein H, Wellner UF, Keck T, Lehnert H, Marquardt JU. RAC1B regulation of TGFB1 reveals an unexpected role of autocrine TGFβ1 in the suppression of cell motility. Cancers (Basel). 2020;12(12):3570. 10.3390/cancers12123570 PMC7760153 33260366

[35] Ungefroren H, Wellner UF, Keck T, Lehnert H, Marquardt JU. The small GTPase RAC1B: a potent negative regulator of-and useful tool to study-TGFβ signaling. Cancers (Basel). 2020;12(11):3475. 10.3390/cancers12113475 PMC7700615 33266416

[36] Sharma P, Zhang X, Ly K, Kim JH, Wan Q, Kim J, et al. Hyperglycosylation of prosaposin in tumor dendritic cells drives immune escape. Science. 2024;383(6679):190–200. 38207022 10.1126/science.adg1955 PMC11398950

[37] Yuan L, Morales CR. A stretch of 17 amino acids in the prosaposin C terminus is critical for its binding to sortilin and targeting to lysosomes. J Histochem Cytochem. 2010;58(3):287–300. 19934382 10.1369/jhc.2009.955203 PMC2825494

[38] Lefrancois S, Zeng J, Hassan AJ, Canuel M, Morales CR. The lysosomal trafficking of sphingolipid activator proteins (SAPs) is mediated by sortilin. EMBO J. 2003;22(24):6430–7. 14657016 10.1093/emboj/cdg629 PMC291824

[39] Kwon S, Christian JL. Sortilin associates with transforming growth factor-beta family proteins to enhance lysosome-mediated degradation. J Biol Chem. 2011;286(24):21876–85. 21521695 10.1074/jbc.M111.228262 PMC3122242

[40] Barnes JW, Aarnio-Peterson M, Norris J, Haskins M, Flanagan-Steet H, Steet R. Upregulation of sortilin, a Lysosomal sorting receptor, corresponds with reduced bioavailability of latent TGFβ in Mucolipidosis II Cells. Biomolecules. 2020;10(5):670. 10.3390/biom10050670 PMC7277838 32357547

[41] Young HH. On the presence of nerves in tumors and of other structures in them as revealed by a modification of ehrlich’s method of “vital staining” with methylene blue. J Exp Med. 1897;2(1):1–12. 19866822 10.1084/jem.2.1.1 PMC2117917

[42] Scherer HJ. Structural development in gliomas. Am J Cancer. 1938;34(3):333–51.

[43] Mohs FE, Lathrop TG. Modes of spread of cancer of skin. AMA Arch Derm Syphilol. 1952;66(4):427–39. 12975852 10.1001/archderm.1952.01530290003001

[44] Shi DD, Guo JA, Hoffman HI, Su J, Mino-Kenudson M, Barth JL, et al. Therapeutic avenues for cancer neuroscience: translational frontiers and clinical opportunities. Lancet Oncol. 2022;23(2):e62–74. 35114133 10.1016/S1470-2045(21)00596-9 PMC9516432

[45] Jessen KR, Mirsky R. Signals that determine Schwann cell identity. J Anat. 2002;200(4):367–76. 12090403 10.1046/j.1469-7580.2002.00046.x PMC1570691

[46] Stierli S, Imperatore V, Lloyd AC. Schwann cell plasticity-roles in tissue homeostasis, regeneration, and disease. Glia. 2019;67(11):2203–15. 31215712 10.1002/glia.23643

[47] Hiraiwa M, Campana WM, Mizisin AP, Mohiuddin L, O’Brien JS. Prosaposin: a myelinotrophic protein that promotes expression of myelin constituents and is secreted after nerve injury. Glia. 1999;26(4):353–60. 10383054

[48] Oya Y, Nakayasu H, Fujita N, Suzuki K, Suzuki K. Pathological study of mice with total deficiency of sphingolipid activator proteins (SAP knockout mice). Acta Neuropathol. 1998;96(1):29–40. 9678511 10.1007/s004010050857

[49] Hiraiwa M, Taylor EM, Campana WM, Darin SJ, O’Brien JS. Cell death prevention, mitogen-activated protein kinase stimulation, and increased sulfatide concentrations in Schwann cells and oligodendrocytes by prosaposin and prosaptides. Proc Natl Acad Sci U S A. 1997;94(9):4778–81. 9114068 10.1073/pnas.94.9.4778 PMC20801

[50] Nodari A, Zambroni D, Quattrini A, Court FA, D’Urso A, Recchia A, et al. Beta1 integrin activates Rac1 in Schwann cells to generate radial lamellae during axonal sorting and myelination. J Cell Biol. 2007;177(6):1063–75. 17576799 10.1083/jcb.200610014 PMC2064366

[51] Benninger Y, Thurnherr T, Pereira JA, Krause S, Wu X, Chrostek-Grashoff A, et al. Essential and distinct roles for cdc42 and rac1 in the regulation of Schwann cell biology during peripheral nervous system development. J Cell Biol. 2007;177(6):1051–61. 17576798 10.1083/jcb.200610108 PMC2064365

[52] Guo L, Moon C, Niehaus K, Zheng Y, Ratner N. Rac1 controls Schwann cell myelination through cAMP and NF2/merlin. J Neurosci. 2012;32(48):17251–61. 23197717 10.1523/JNEUROSCI.2461-12.2012 PMC3601465

[53] Yan L, Wen Z, Yang Y, Liu A, Li F, Zhang Y, et al. Dissecting the roles of prosaposin as an emerging therapeutic target for tumors and its underlying mechanisms. Biomed Pharmacother. 2024;180:117551. 39405903 10.1016/j.biopha.2024.117551

[54] Peipp M, Dudziak D, Kellner C. Prosaposin hyperglycosylation: a novel tumor immune escape mechanism and implications for cancer immunotherapy. Signal Transduct Target Ther. 2024;9(1):172. 38982080 10.1038/s41392-024-01877-2 PMC11233631

[55] Tatti M, Motta M, Di Bartolomeo S, Cianfanelli V, Salvioli R. Cathepsin-mediated regulation of autophagy in saposin C deficiency. Autophagy. 2013;9(2):241–3. 23108186 10.4161/auto.22557 PMC3552889

[56] Tayebi N, Lopez G, Do J, Sidransky E. Pro-cathepsin D, Prosaposin, and Progranulin: Lysosomal networks in parkinsonism. Trends Mol Med. 2020;26(10):913–23. 32948448 10.1016/j.molmed.2020.07.004 PMC9067398

[57] Jeong MH, Park SY, Lee SH, Seo J, Yoo JY, Park SH, et al. EPB41L5 mediates TGFβ-induced metastasis of gastric cancer. Clin Cancer Res. 2019;25(12):3617–29. 30814110 10.1158/1078-0432.CCR-18-2959

[58] Xie L, Qiu S, Lu C, Gu C, Wang J, Lv J, et al. Gastric cancer-derived LBP promotes liver metastasis by driving intrahepatic fibrotic pre-metastatic niche formation. J Exp Clin Cancer Res. 2023;42(1):258. 37789385 10.1186/s13046-023-02833-8 PMC10546721

[59] Shimura M, Matsuo J, Pang S, Jangphattananont N, Hussain A, Rahmat MB, et al. Iqgap3 signalling mediates intratumoral functional heterogeneity to enhance malignant growth. Gut. 2025;74(3):364–86. 39438124 10.1136/gutjnl-2023-330390 PMC11874294

[60] Vaes N, Idris M, Boesmans W, Alves MM, Melotte V. Nerves in gastrointestinal cancer: from mechanism to modulations. Nat Rev Gastroenterol Hepatol. 2022;19(12):768–84. 36056202 10.1038/s41575-022-00669-9

[61] Jiang Y, Zhou J, Luo P, Gao H, Ma Y, Chen YS, et al. Prosaposin promotes the proliferation and tumorigenesis of glioma through toll-like receptor 4 (TLR4)-mediated NF-κB signaling pathway. EBioMedicine. 2018;37:78–90. 30385233 10.1016/j.ebiom.2018.10.053 PMC6286187

[62] Jiang Y, Zhou J, Hou D, Luo P, Gao H, Ma Y, et al. Prosaposin is a biomarker of mesenchymal glioblastoma and regulates mesenchymal transition through the TGF-β1/Smad signaling pathway. J Pathol. 2019;249(1):26–38. 30953361 10.1002/path.5278

[63] Miyahara Y, Takano S, Sogawa K, Tomizawa S, Furukawa K, Takayashiki T, et al. Prosaposin, tumor-secreted protein, promotes pancreatic cancer progression by decreasing tumor-infiltrating lymphocytes. Cancer Sci. 2022;113(8):2548–59. 35633503 10.1111/cas.15444 PMC9357616

[64] Shi X, Lingerak R, Herting CJ, Ge Y, Kim S, Toth P, et al. Time-resolved live-cell spectroscopy reveals EphA2 multimeric assembly. Science. 2023;382(6674):1042–50. 37972196 10.1126/science.adg5314 PMC11114627

[65] Mason J, Öhlund D. Key aspects for conception and construction of co-culture models of tumor-stroma interactions. Front Bioeng Biotechnol. 2023;11:1150764. 37091337 10.3389/fbioe.2023.1150764 PMC10119418

