# Supplementary material for: Prosaposin orchestrates a TGFβ1-driven paracrine loop between Schwann cells and gastric cancer to accelerate perineural invasion
Source: J Exp Clin Cancer Res. 2026 Jan 24;45:56. doi: 10.1186/s13046-026-03652-3 (PMC12911069; doi:10.1186/s13046-026-03652-3)
Supplement: Supplementary file 1 — Supplementary Material 1. [file 13046_2026_3652_MOESM1_ESM.docx]

| **Gene name (Species information)**  **Supplementary Table 1.** | **Sequence** | | | |
| --- | --- | --- | --- | --- |
|  | **sense（5'-3'）** | **antisense（5'-3'）** | | |
| PSAP-Homo-sh1 | CTTCCTTCGTGTGCTTGAA | TTCAAGCACACGAAGGAAG | | |
| PSAP-Homo-sh2 | GCCGACATATGCAAGAACT | AGTTCTTCGCATATGTCGGC | | |
| PSAP-Homo-sh3 | CCAAGCTGATTGACAACAA | TTGTTGTCAATCAGCTTGG | | |
| PSAP- homo  LV5(EF-1a/GFP&Puro) | ATGTACGCCCTCTTCCTCCTGGCCAGCCTCCTGGGCGCGGCTCTAGCCGGCCCGGTCCTTGGACTGAAAGAATGCACCAGGGGCTCGGCAGTGTGGTGCCAGAATGTGAAGACGGCGTCCGACTGCGGGGCAGTGAAGCACTGCCTGCAGACCGTTTGGAACAAGCCAACAGTGAAATCCCTTCCCTGCGACATATGCAAAGACGTTGTCACCGCAGCTGGTGATATGCTGAAGGACAATGCCACTGAGGAGGAGATCCTTGTTTACTTGGAGAAGACCTGTGACTGGCTTCCGAAACCGAACATGTCTGCTTCATGCAAGGAGATAGTGGACTCCTACCTCCCTGTCATCCTGGACATCATTAAAGGAGAAATGAGCCGTCCTGGGGAGGTGTGCTCTGCTCTCAACCTCTGCGAGTCTCTCCAGAAGCACCTAGCAGAGCTGAATCACCAGAAGCAGCTGGAGTCCAATAAGATCCCAGAGCTGGACATGACTGAGGTGGTGGCCCCCTTCATGGCCAACATCCCTCTCCTCCTCTACCCTCAGGACGGCCCCCGCAGCAAGCCCCAGCCAAAGGATAATGGGGACGTTTGCCAGGACTGCATTCAGATGGTGACTGACATCCAGACTGCTGTACGGACCAACTCCACCTTTGTCCAGGCCTTGGTGGAACATGTCAAGGAGGAGTGTGACCGCCTGGGCCCTGGCATGGCCGACATATGCAAGAACTATATCAGCCAGTATTCTGAAATTGCTATCCAGATGATGATGCACATGCAACCCAAGGAGATCTGTGCGCTGGTTGGGTTCTGTGATGAGGTGAAAGAGATGCCCATGCAGACTCTGGTCCCCGCCAAAGTGGCCTCCAAGAATGTCATCCCTGCCCTGGAACTGGTGGAGCCCATTAAGAAGCACGAGGTCCCAGCAAAGTCTGATGTTTACTGTGAGGTGTGTGAATTCCTGGTGAAGGAGGTGACCAAGCTGATTGACAACAACAAGACTGAGAAAGAAATACTCGACGCTTTTGACAAAATGTGCTCGAAGCTGCCGAAGTCCCTGTCGGAAGAGTGCCAGGAGGTGGTGGACACGTACGGCAGCTCCATCCTGTCCATCCTGCTGGAGGAGGTCAGCCCTGAGCTGGTGTGCAGCATGCTGCACCTCTGCTCTGGCACGCGGCTGCCTGCACTGACCGTTCACGTGACTCAGCCAAAGGACGGTGGCTTCTGCGAAGTGTGCAAGAAGCTGGTGGGTTATTTGGATCGCAACCTGGAGAAAAACAGCACCAAGCAGGAGATCCTGGCTGCTCTTGAGAAAGGCTGCAGCTTCCTGCCAGACCCTTACCAGAAGCAGTGTGATCAGTTTGTGGCAGAGTACGAGCCCGTGCTGATCGAGATCCTGGTGGAGGTGATGGATCCTTCCTTCGTGTGCTTGAAAATTGGAGCCTGCCCCTCGGCCCATAAGCCCTTGTTGGGAACTGAGAAGTGTATATGGGGCCCAAGCTACTGGTGCCAGAACACAGAGACAGCAGCCCAGTGCAATGCTGTCGAGCATTGCAAACGCCATGTGTGGAACTAG | | | |
| GPR37-Rattus-sh1 | GTTGTGACCGAAAGTACATTG | | | CAATGTACTTTCGGTCACAAC |
| GPR37-Rattus-sh2 | GTACACGAAGGAAGAACCCTT | | | AAGGGTTCTTCCTTCGTGTAC |
| GPR37-Rattus-sh3 | GAACCTGAACCCAGAGATCTT | | | AAGATCTCTGGGTTCAGGTTC |
| Sortilin- homo  LV5(EF-1a/GFP&Puro) | ACCGGTGCCACCATGGAGCGGCCCTGGGGAGCTGCGGACGGCCTCTCGCGCTGGCCCCATGGCCTCGGCCTCCTCCTCCTCCTGCAGCTGCTGCCGCCGTCGACCCTCAGCCAGGACCGGCTGGACGCGCCGCCGCCGCCCGCTGCGCCGCTGCCGCGCTGGTCTGGCCCCATCGGGGTGAGCTGGGGGCTGCGGGCGGCCGCAGCCGGG  GGCGCGTTTCCCGCGGCGGCCGTTGGCGTCGCAGCGCGCCGGGCGAGGACGAGGAGTGCGGCCGGGTCCGGGACTTCGTCGCCAAGCTGGCCAACAACACGCACCAGCATGTGTTTGATGATCTCAGAGGCTCAGTATCCTTGTCCTGGGTTGGAGATAGCACTGGGGTCATTCTAGTCTTGACTACCTTCCATGTACCACTGGTAATTATGACTTTTGGACAGTCCAAGCTATATCGAAGTGAGGATTATGGGAAGAACTTTAAGGATATTACAGATCTCATCAATAACACCTTTATTCGGACTGAATTTGGCATGGCTATTGGTCCTGAGAACTCTGGAAAGGTGGTGTTAACAGCAGAGGTGTCTGGAGGAAGTCGTGGAGGAAGAATCTTTAGATCATCAGATTTTGCGAAGAATTTTGTGCAAACAGATCTCCCTTTTCATCCTCTCACTCAGATGATGTATAGCCCTCAGAATTCTGATTATCTTTTAGCTCTCAGCACTGAAAATGGCCTGTGGGTGTCCAAGAATTTTGGGGGAAAATGGGAAGAAATCCACAAAGCAGTATGTTTGGCCAAATGGGGATCAGACAACACCATCTTCTTTACAACCTATGCAAATGGCTCCTGCAAAGCTGACCTTGGGGCTCTGGAATTATGGAGAACTTCAGACTTGGGAAAAAGCTTCAAAACTATTGGTGTGAAAATCTACTCATTTGGTCTTGGGGGACGTTTCCTTTTTGCCTCTGTGATGGCTGATAAGGATACAACAAGAAGGATCCACGTTTCAACAGATCAAGGGGACACATGGAGCATGGCCCAGCTCCCCTCCGTGGGACAGGAACAGTTCTATTCTATTCTGGCAGCAAATGATGACATGGTATTCATGCATGTAGATGAACCTGGAGACACTGGGTTTGGCACAATCTTTACCTCAGATGATCGAGGCATTGTCTATTCCAAGTCTTTGGACCGACATCTCTACACTACCACAGGCGGAGAGACGGACTTTACCAACGTGACCTCCCTCCGCGGCGTCTACATAACAAGCGTGCTCTCCGAAGATAATTCTATCCAGACCATGATCACTTTTGACCAAGGAGGAAGGTGGACGCACCTGAGGAAGCCTGAAAACAGTGAATGTGATGCTACAGCAAAAAACAAGAATGAGTGCAGCCTTCATATTCATGCTTCCTACAGCATCTCCCAGAAACTGAATGTTCCAATGGCCCCACTCTCAGAGCCGAATGCCGTAGGCATTGTCATTGCTCATGGTAGCGTGGGGGATGCCATCTCAGTGATGGTTCCAGATGTGTACATCTCAGATGATGGGGGTTACTCCTGGACAAAGATGCTGGAAGGACCCCACTATTACACCATCCTGGATTCTGGAGGCATCATTGTGGCCATTGAGCACAGCAGCCGTCCTATCAATGTGATTAAGTTCTCCACAGACGAAGGTCAATGCTGGCAAACCTACACGTTCACCAGGGACCCCATCTATTTCACTGGCCTAGCTTCAGAACCTGGAGCTAGGTCCATGAATATCAGCATTTGGGGCTTCACAGAATCTTTCCTGACCAGCCAGTGGGTCTCCTACACCATTGATTTTAAAGATATCCTTGAAAGGAACTGTGAAGAGAAGGACTATACCATATGGCTGGCACACTCCACAGACCCTGAAGATTATGAAGATGGCTGCATTTTGGGCTACAAAGAACAGTTTCTGCGGCTACGCAAGTCATCCGTGTGTCAGAATGGTCGAGACTATGTTGTGACCAAGCAGCCCTCCATCTGCCTCTGTTCCCTGGAGGACTTTCTCTGTGATTTTGGCTACTACCGTCCAGAAAATGACTCCAAGTGTGTGGAACAGCCAGAACTGAAGGGCCACGACCTGGAGTTTTGTCTGTACGGAAGAGAAGAACACCTAACAACAAATGGGTACCGGAAAATTCCAGGGGACAAATGCCAGGGTGGGGTAAATCCAGTTCGAGAAGTAAAAGACTTGAAAAAGAAATGCACAAGCAACTTTTTGAGTCCGGAAAAACAGAATTCCAAGTCAAATTCTGTTCCAATTATCCTGGCCATCGTGGGATTGATGCTGGTCACAGTCGTAGCAGGAGTGCTCATTGTGAAGAAATATGTCTGTGGGGGAAGGTTCCTGGTGCATCGATACTCTGTGCTGCAGCAGCATGCAGAGGCCAATGGTGTGGATGGTGTGGATGCTTTGGACACAGCCTCCCACACTAATAAAAGTGGTTATCATGATGACTCAGATGAGGACCTCTTGGAATAGGAATTC | | |  |
| Rescue-GPR37-Rattus-sh2 | Region: NM_057201.2  RefSeq position (1-based): 1325–1345  Original 21-nt (sense): GTACACGAAGGAAGAACCCTT  Recoded 21-nt for ORF-only: GTCCATGAGGGCCGCACATTA  Peptide (7 aa): V-H-E-G-R-T-L | | | |
| TGFβ1- Rattus -siRNA-1 | UGUCCAAACUAAGGCUCGC | | ACAGGUUUGAUUCCGAGCG | |
| TGFβ1- Rattus -siRNA-2 | GCUCGCUUUGUACAACAGC | | CGAGCGAAACAUGUUGUCG | |
| TGFβ1- Rattus -siRNA-3 | GAACCAAGGAGACGGAAUA | | CUUGGUUCCUCUGCCUUAU | |
| **Primer names** | **Primer sequence** | | | |
| sortilin promoter-WT-Top | AAACTAGCGGCCGCTAGTTTTGCTAAGTCTGGAAATCATGT | | | |
| sortilin promoter-WT-Bot | CTAGACATGATTTCCAGACTTAGCAAAACTAGCGGCCGCTAGTTT | | | |
| sortilin promoter-Mut-Top | AAACTAGCGGCCGCTAGTTTTGCTAGCAGACCTAATCATGT | | | |
| sortilin promoter-Mut-Bot | CTAGACATGATTAGGTCTGCTAGCAAAACTAGCGGCCGCTA GTTT | | | |
| sortilin promoter-Top | GATGCTGGCTTCTGGAGATAAGAAGGT | | | |
| sortilin promoter-Bot | TGAAACAAGGGATGATGCCATGAT | | | |
| **Sequencing results of dual luciferase reporter plasmids** | | | | |
| **>Sortilin promoter-WT:**  GGGGAAGGGCATGTGGCCAGCCAGGTTACAACCGCCAAGAAGCTGCGCGGTGGTGTTGTGTTCGTGGACGAGGTGCCTAAAGGACTGACCGGCAAGTTGGACGCCCGCAAGATCCGCGAGATTCTCATTAAGGCCAAGAAGGGCGGCAAGATCGCCGTGTAATTCTAGTTGTTTAAACTAGCGGCCGCTAGTTTTGCTAAGTCTGGAAATCATGTCTAGAGTCGACCTGCAGGCATGCAAGCTGATCCGGCTGCTAACAAAGCCCGAAAGGAAGCTGAGTTGGCTGCTGCCACCGCTGAGCAATAACTAGCATAACCCCTTGGGGCGGCCGCTTCGAGCAGACATGATAAGATACATTGATGAGTTTGGACAAACCACAACTAGAATGCAGTGAAAAAAATGCTTTATTTGTGAAATTTGTGATGCTATTGCTTTATTTGTAACCATTATAAGCTGCAATAAACAAGTTAACAACAACAATTGCATTCATTTTATGTTTCAGGTTCAGGGGGAGATGTGGGAGGTTTTTTTAAGCAAGTAAAACCTCTACAAATGTGGTAAAATCGAATTTTAACAAAATATTAACGCTTACAATTTCCTGATGCGGTATTTTCTCCTTACGCATCTGTGCGGTATTTCACACCGCATACGCGGATCTGCGCAGCACCATGGCCTGAAATAACCTCTGAAAGAGGAACTTGGTTAGGTACCTTCTGAGGCGGAAAGAACCAGCTGTGGAATGTGTGTCAGTTAGGGTGTGGAAAGTCCCCAGGCTCCCCAGCAGGCAGAAGTATGCAAAGCATGCATCTCAATTAGTCAGCAACCAGGTGTGGAAAGTCCCCAGGCTCCCCAGCAGGCAGAAGTATGCAAAGCATGCATCTCATTATCAAGCAACCATAGTCCCGCCCCTAACTCCGCCCATCCCGCCCTAACTCCGCCAGTTCCGCCATTCTCGCCCCAGGGCTGACAAATT  **>Sortilin promoter-Mut**  GGGGAAGGGAATGTGGCCAGCCAGGTTACAACCGCCAAGAAGCTGCGCGGTGGTGTTGTGTTCGTGGACGAGGTGCCTAAAGGACTGACCGGCAAGTTGGACGCCCGCAAGATCCGCGAGATTCTCATTAAGGCCAAGAAGGGCGGCAAGATCGCCGTGTAATTCTAGTTGTTTAAACTAGCGGCCGCTAGTTTTGCTAGCAGACCTAATCATGTCTAGAGTCGACCTGCAGGCATGCAAGCTGATCCGGCTGCTAACAAAGCCCGAAAGGAAGCTGAGTTGGCTGCTGCCACCGCTGAGCAATAACTAGCATAACCCCTTGGGGCGGCCGCTTCGAGCAGACATGATAAGATACATTGATGAGTTTGGACAAACCACAACTAGAATGCAGTGAAAAAAATGCTTTATTTGTGAAATTTGTGATGCTATTGCTTTATTTGTAACCATTATAAGCTGCAATAAACAAGTTAACAACAACAATTGCATTCATTTTATGTTTCAGGTTCAGGGGGAGATGTGGGAGGTTTTTTTAAGCAAGTAAAACCTCTACAAATGTGGTAAAATCGAATTTTAACAAAATATTAACGCTTACAATTTCCTGATGCGGTATTTTCTCCTTACGCATCTGTGCGGTATTTCACACCGCATACGCGGATCTGCGCAGCACCATGGCCTGAAATAACCTCTGAAAGAGGAACTTGGTTAGGTACCTTCTGAGGCGGAAAGAACCAGCTGTGGAATGTGTGTCAGTTAGGGTGTGGAAAGTCCCCAGGCTCCCCAGCAGGCAGAAGTATGCAAAGCATGCATCTCAATTAGTCAGCAACCAGGTGTGGAAAGTCCCCAGGCTCCCCAGCAGGCAGAAGTATGCAAAGCATGCATCTCATTAGTCAAGCAACCATAGTCCCGCCCCTAAATCCGCCCATCCCGCCCCTAACTCCGCCCAGTTCCGCCCATTCTCCGCCCAGGGCTGACCAT | | | | |

**Supplementary Table 2**.

| **Antibody** | **Multiple of dilution** | **Item number** | **Brand (Origin)** | **Purpose** |
| --- | --- | --- | --- | --- |
| anti-PGP9.5 | 1:500 | #GB12159 | Servicebio,China | IHC/mIHC |
| anti-S100β | 1:200 | #GB11359 | Servicebio,China | IHC/mIHC |
| anti-CK7 | 1:200 | #GB115696] | Servicebio,China | IHC/mIHC |
| anti-PSAP | 1:500 | #GB111172 | Servicebio,China | IHC/mIHC |
| anti-TGFβ1 | 1:200 | #GB111876 | Servicebio,China | IHC/mIHC |
| anti-PSAP | 1:5000 | #10801-1-AP | Proteintech,China | WB |
| anti-TGFβ1 | 1:2000 | # 26155-1-AP | Proteintech,China | WB |
| antiGPR37 | 1:1000 | # ER63602 | HUABIO,China | WB |
| antiRAC1 | 1:2000 | #24072-1-AP | Proteintech,China | WB |
| antiACTB | 1:8000 | #20536-1-AP | Proteintech,China | WB |
| antiSmad4 | 1:4000 | # ET1604-12 | HUABIO,China | WB |
| antiSmad4 | 1:5000 | #AF2097-SP | R&D Systems,USA | Chip |
| antiTGFβRⅡ | 1:1000 | # HA721693 | HUABIO,China | WB |
| antiSortilin | 1:1000 | #R381897 | ZenBIO,China | WB |
| antiCTSD | 1:1000 | # R380946 | ZenBIO,China | WB |
| antiGALC | 1:1000 | # HA722004 | HUABIO,China | WB |
| antiGAPDH | 1:5000 | #GB15004-100 | Servicebio,China | WB |
| antiTublin | 1:5000 | #GB15140-100 | Servicebio,China | WB |
| Goat anti-mouse IgG -HRP | 1:50000 | # HA1006 | HUABIO,China | WB |
| Goat anti-rabbit IgG -HRP | 1:50000 | # HA1001 | HUABIO,China | WB |
|  |  |  |  |  |
|  |  |  |  |  |
