# Supplementary material for: Prosaposin orchestrates a TGFβ1-driven paracrine loop between Schwann cells and gastric cancer to accelerate perineural invasion
Source: J Exp Clin Cancer Res. 2026 Jan 24;45:56. doi: 10.1186/s13046-026-03652-3 (PMC12911069; doi:10.1186/s13046-026-03652-3)

Supplementary Figure1

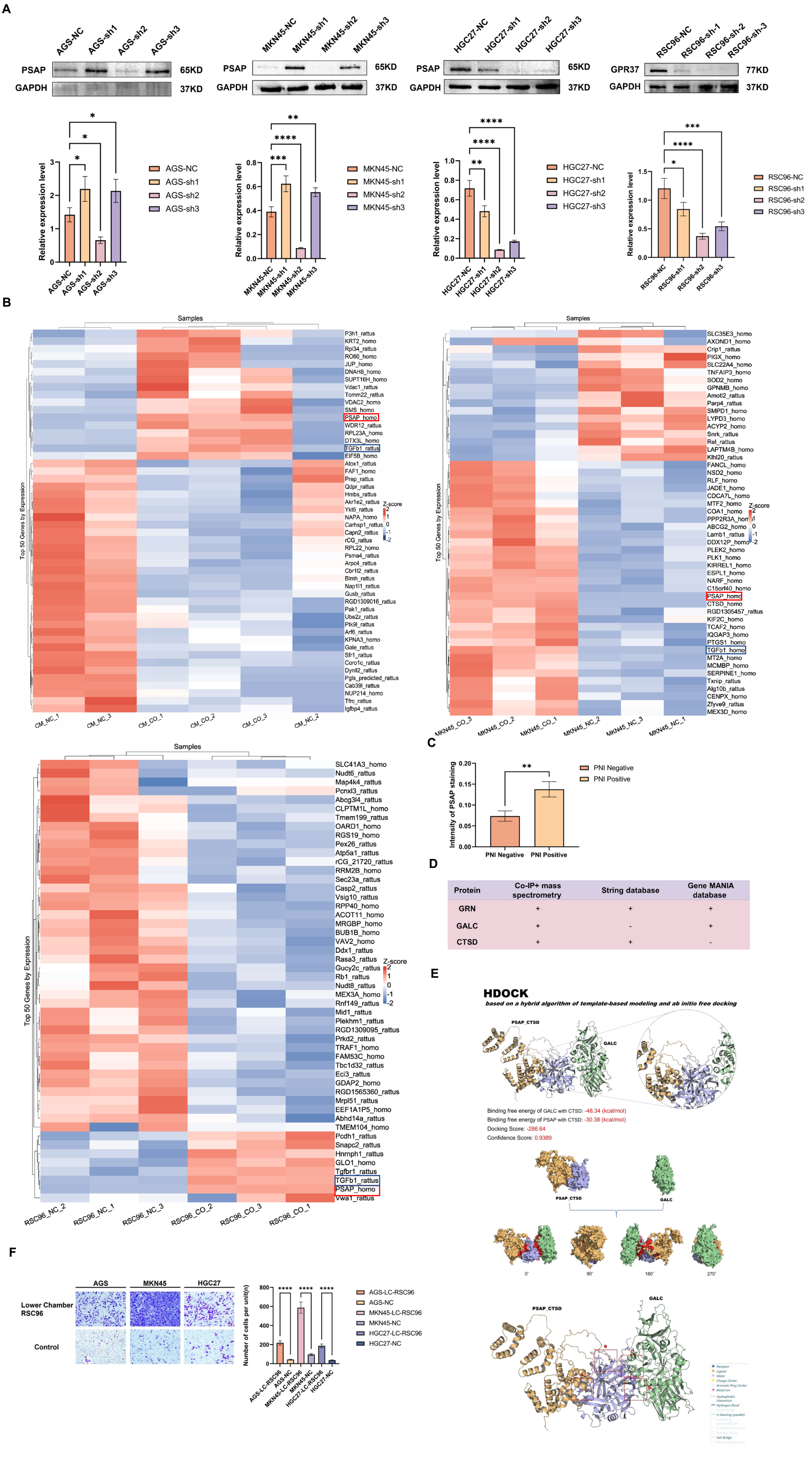

Protein	Co-IP+ mass spectrometry	String database	Gene MANIA database
GRN	+	+	+
GALC	+	-	+
CTSD	+	+	-

Supplementary Figure1

G

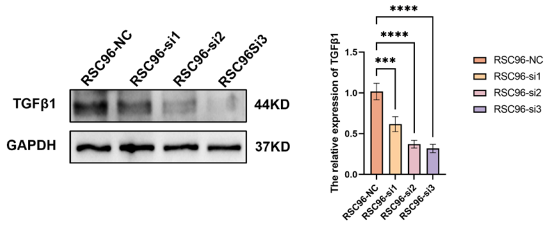

H

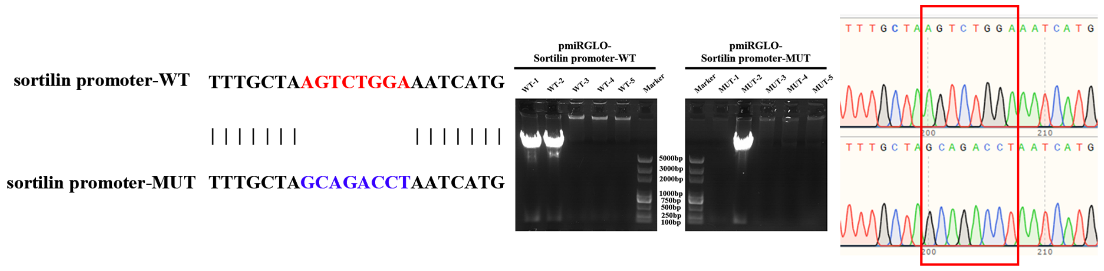

I

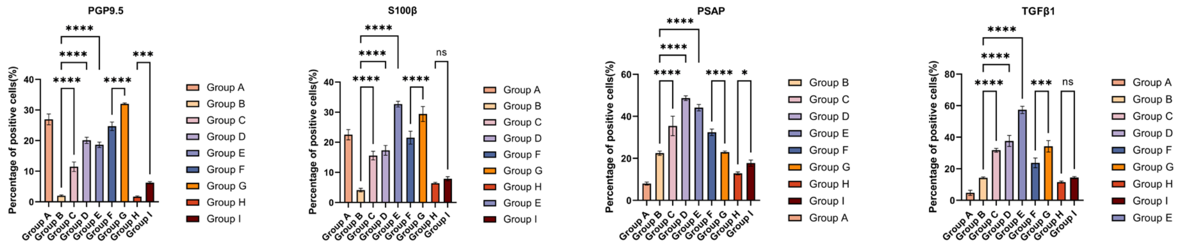

Supplementary Figure2

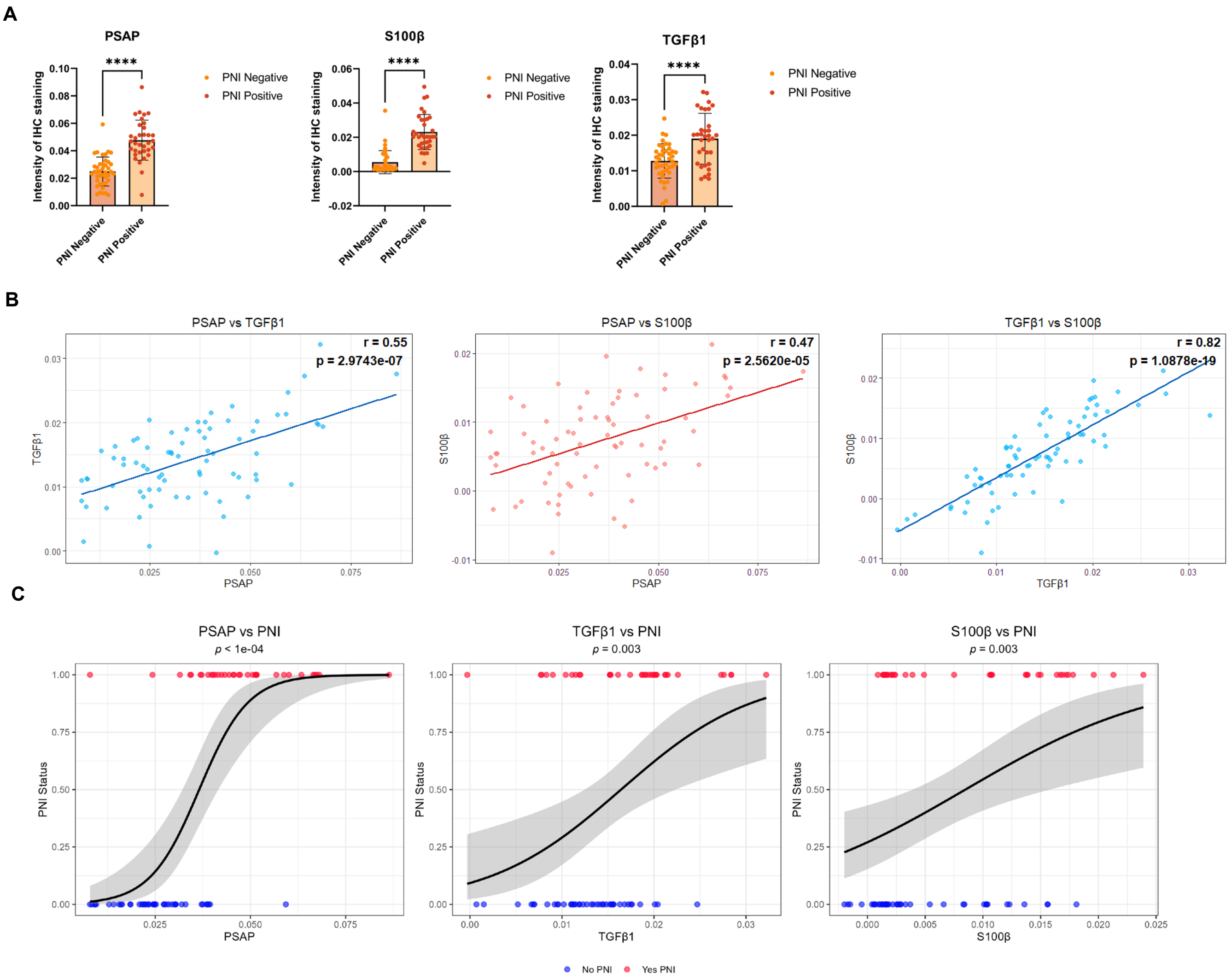

Supplement: Supplementary file 2 — Supplementary Material 2. [file 13046_2026_3652_MOESM2_ESM.pdf]
